# Mendelian randomization analysis of 37 clinical factors and coronary artery disease in East Asian and European populations

**DOI:** 10.1186/s13073-022-01067-1

**Published:** 2022-06-14

**Authors:** Kai Wang, Xian Shi, Ziwei Zhu, Xingjie Hao, Liangkai Chen, Shanshan Cheng, Roger S. Y. Foo, Chaolong Wang

**Affiliations:** 1 Department of Epidemiology and Biostatistics, Ministry of Education Key Laboratory of Environment and Health, School of Public Health, Tongji Medical College, Huazhong University of Science and Technology, Wuhan, China; 2 Department of Nutrition and Food Hygiene, Hubei Key Laboratory of Food Nutrition and Safety, School of Public Health, Tongji Medical College, Huazhong University of Science and Technology, Wuhan, China; 3 Cardiovascular Research Institute, Centre for Translational Medicine, National University Health System, Singapore, Singapore; 4 Genome Institute of Singapore, Singapore, Singapore

**Keywords:** Mendelian randomization, Clinical factors, Coronary artery disease, Causal inference, Red blood cells, Uric acid

## Abstract

**Background:**

Coronary artery disease (CAD) remains the leading cause of mortality worldwide despite enormous efforts devoted to its prevention and treatment. While many genetic loci have been identified to associate with CAD, the intermediate causal risk factors and etiology have not been fully understood. This study assesses the causal effects of 37 heritable clinical factors on CAD in East Asian and European populations.

**Methods:**

We collected genome-wide association summary statistics of 37 clinical factors from the Biobank Japan (42,793 to 191,764 participants) and the UK Biobank (314,658 to 442,817 participants), paired with summary statistics of CAD from East Asians (29,319 cases and 183,134 controls) and Europeans (91,753 cases and 311,344 controls). These clinical factors covered 12 cardiometabolic traits, 13 hematological indices, 7 hepatological and 3 renal function indices, and 2 serum electrolyte indices. We performed univariable and multivariable Mendelian randomization (MR) analyses in East Asians and Europeans separately, followed by meta-analysis.

**Results:**

Univariable MR analyses identified reliable causal evidence (*P* < 0.05/37) of 10 cardiometabolic traits (height, body mass index [BMI], blood pressure, glycemic and lipid traits) and 4 other clinical factors related to red blood cells (red blood cell count [RBC], hemoglobin, hematocrit) and uric acid (UA). Interestingly, while generally consistent, we identified population heterogeneity in the causal effects of BMI and UA, with higher effect sizes in East Asians than those in Europeans. After adjusting for cardiometabolic factors in multivariable MR analysis, red blood cell traits (RBC, meta-analysis odds ratio 1.07 per standard deviation increase, 95% confidence interval 1.02–1.13; hemoglobin, 1.10, 1.03–1.16; hematocrit, 1.10, 1.04–1.17) remained significant (*P* < 0.05), while UA showed an independent causal effect in East Asians only (1.12, 1.06–1.19, *P* = 3.26×10^−5^).

**Conclusions:**

We confirmed the causal effects of 10 cardiometabolic traits on CAD and identified causal risk effects of RBC, hemoglobin, hematocrit, and UA independent of traditional cardiometabolic factors. We found no causal effects for 23 clinical factors, despite their reported epidemiological associations. Our findings suggest the physiology of red blood cells and the level of UA as potential intervention targets for the prevention of CAD.

**Supplementary Information:**

The online version contains supplementary material available at 10.1186/s13073-022-01067-1.

## Background

Coronary artery disease (CAD) is the foremost cause of mortality worldwide. In 2019, CAD was estimated to affect 197 million patients globally and accounted for 9.1 million deaths (16.1% of all deaths) [1, 2]. CAD has a high heritability and often develops over decades before a symptomatic ischemia or any acute coronary event. Early intervention is essential to reduce the morbidity and mortality of CAD, which would have far-reaching implications for the related public health burden. However, CAD has a complex etiology involving the interplay of genetic and environmental factors [3]. Identification of causal risk factors is important for early precision prevention. In particular, improved understanding of the causality and effect sizes of different risk factors can refine prevention strategies and enable novel therapeutic targets for CAD.

To date, although hundreds of risk factors are being reported to associate with CAD by epidemiological studies [4], causal inference of these associations was hindered by unmeasured confounding and reverse causation. For instance, despite a strong association between circulating levels of lipoprotein-associated phospholipase A2 and the risk of coronary events [5], their causality was not verified by randomized controlled trials (RCTs) [6, 7]. The recent advances of large-scale genome-wide association studies (GWAS) and Mendelian randomization (MR) methods have enabled evaluation of the causality between risk factors and disease outcomes [8]. In particular, MR analyses have uncovered the causal roles of height [9], body mass index (BMI) [10], serum lipids [11], blood pressure [12], hemoglobin A1c (HbA1c) [13], and type 2 diabetes (T2D) [13] in CAD. Yet, only one third of the variants associated with CAD mediate through the well-known cardiometabolic risk factors, such as lipids and blood pressure [14]. These findings suggested the existence of other causal pathways, which might provide novel insights into the etiology of CAD. Furthermore, the reported MR evidence were largely derived from European samples, and the generalizability to non-Europeans remains unverified due to different environmental background between populations.

In this study, we investigated and compared the causal effects of 37 clinical factors on CAD in East Asian and European populations. We developed a unified MR analysis framework to analyze GWAS summary statistics from the Biobank Japan (BBJ) [15–17], the UK Biobank (UKB) [18, 19], the Coronary Artery Disease Genome-Wide Replication and Meta-analysis plus the Coronary Artery Disease Genetics (CARDIoGRAMplusC4D) consortium [20], and the FinnGen study [21]. These clinical factors included cardiometabolic, hematological, hepatic and renal function related, as well as serum electrolyte factors. Finally, we searched for clinical factors independent of cardiometabolic pathways by applying multivariable MR (MVMR) analysis with adjustment for traditional cardiometabolic factors.

## Methods

### Datasets

To conduct MR analysis, we collected GWAS summary statistics of CAD and clinical factors from the largest publicly available datasets of East Asian and European populations. For East Asians, we collected summary statistics of clinical factors from BBJ, a patient-based biobank with ~200,000 participants recruited from 12 medical institutions across Japan in 2003–2008 [15, 16]. Summary statistics of CAD in East Asians were from Ishigaki et al. [17], consisting of 29,319 cases and 183,134 controls primarily from BBJ. CAD in Ishigaki et al. [17] included physician-diagnosed stable angina, unstable angina, and myocardial infarction (MI). For Europeans, we meta-analyzed CAD GWAS summary statistics from the CARDIoGRAMplusC4D consortium [20] and the FinnGen study [21] using the inverse-variance-weighted (IVW) fixed-effect model [22]. The FinnGen study (release 5) involved 30,952 cases and 187,840 controls of Finnish ancestry [21], with CAD determined by the International Classification of Diseases version 10 (ICD-10) [23], including angina (I20), MI (I21, I22), complications following MI (I23), status post-acute MI (I253), coronary atherosclerosis (I24, I25, Z951, T822), and coronary revascularization. The CARDIoGRAMplusC4D involved 60,801 cases and 123,504 controls from 48 contributing studies, in which CAD included chronic stable angina, MI, acute coronary syndrome, and coronary stenosis >50% [20]. The majority of the CARDIoGRAMplusC4D samples were Europeans (77%), with the rest including South Asians (13%), East Asians (6%), and other ancestries. The European GWAS of clinical factors were based on UKB, a population cohort with over 500,000 participants recruited from 22 assessment centers throughout the UK in 2006–2010 [18]. We used GWAS summary statistics of clinical factors based on 361,194 white-British participants released by the Neale Lab (http://www.nealelab.is/uk-biobank) [24], except for T2D, which were from Mahajan et al. [19], including 18,197 T2D cases and 424,620 controls from UKB. We also obtained individual phenotype data of UKB participants to assess phenotypic correlations, as well as mean and standard deviation (SD) of quantitative clinical factors.

### Study design

**Fig. 1:**
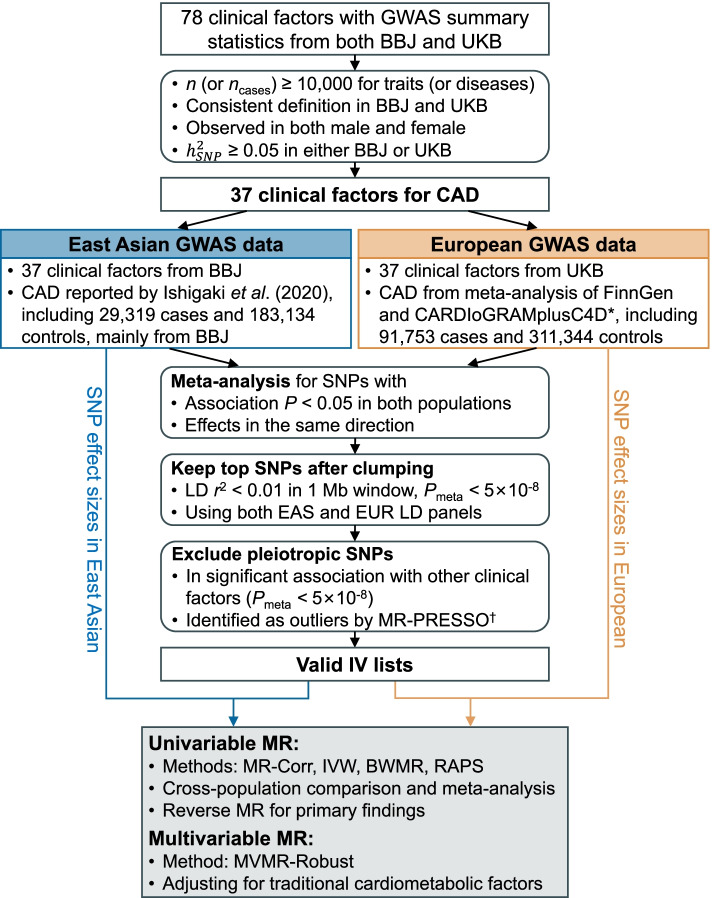
Flowchart of the data collection, processing, and analysis procedures of this study. ^*^ The CARDIoGRAMplusC4D study consisted of primarily Europeans (77%) but also included non-Europeans (13% South Asians, 6% East Asians, and others), ^†^ MR-PRESSO was performed to identify pleiotropic IVs based on GWAS summary statistics from each population

Figure 1 illustrates the overall study design. First, we reviewed and selected 37 clinical factors with GWAS data available in BBJ and UKB. Second, we chose instrumental variables (IVs) for each clinical factor based on a meta-analysis of summary statistics from BBJ and UKB. Third, we performed a univariable MR (UVMR) analysis to estimate population-specific causal effects of each clinical factor on CAD in East Asians and Europeans using four established methods (refer to Methods further below), followed by meta-analysis. Finally, we investigated causal effects independent of traditional cardiometabolic pathways by MVMR.

### Selection of clinical factors for CAD

**Fig. 2:**
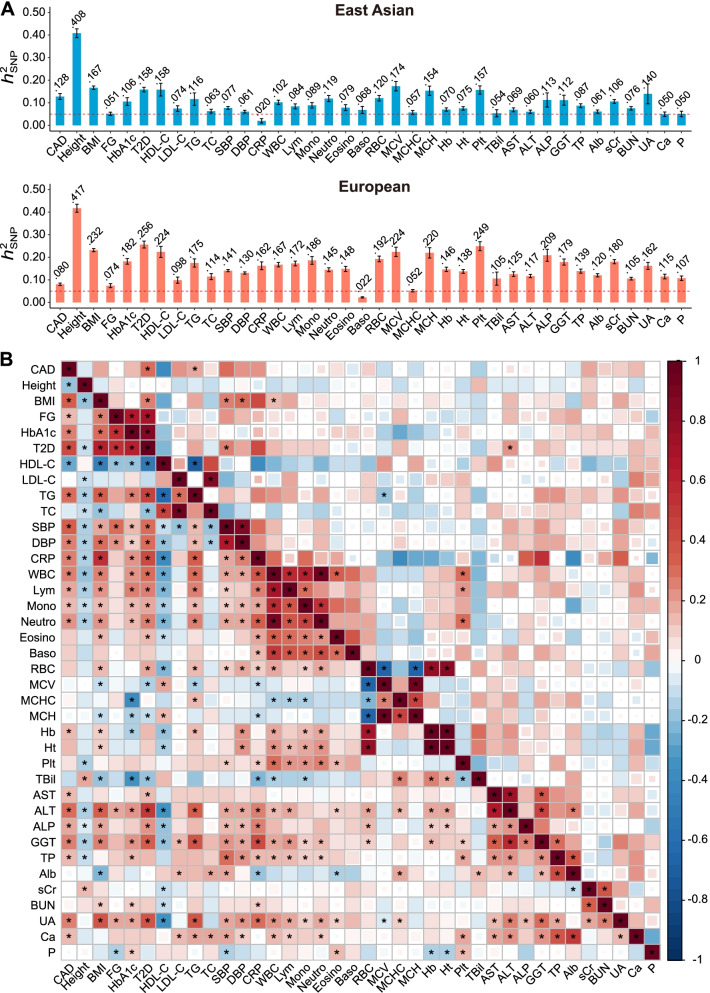
Heritability and genetic correlation for 37 clinical factors and CAD estimated by LDSC. **A** Estimated SNP heritability for each trait/disease in East Asians and Europeans. The error bar indicates one SE and the dotted horizontal line indicates heritability cutoff of 0.05. **B** Genetic correlations in East Asians (upper triangular) and Europeans (lower triangular). Size of the square corresponds to the statistical significance of genetic correlation, and those with *P* < 0.05 are shown in full size. Genetic correlations that are significant after Bonferroni correction (*P* < 0.05/703, where 703 = 38×37/2) are marked with an asterisk

There are 120 traits in BBJ and 4178 traits in UKB, of which GWAS summary statistics are publicly available. We first identified 78 traits in common across the BBJ and UKB GWAS databases. We then excluded 27 disease traits with case sample size *n*_cases_ < 10,000 in BBJ or UKB, 12 traits with inconsistent definitions in BBJ and UKB, and 2 female-specific traits (Additional file 1: Fig. S1). We computed the single nucleotide polymorphism (SNP)-based heritability (${h}_{\mathrm{SNP}}^2$) for each trait by linkage-disequilibrium score regression (LDSC) [25], and found all 37 remaining traits had ${h}_{\mathrm{SNP}}^2$ > 0.05 in at least one population (Fig. 2). These 37 traits include 12 cardiometabolic, 13 hematological, 7 hepatic and 3 renal function related, and 2 serum electrolyte factors, all of which, except for mean corpuscular hemoglobin (MCH) and total protein (TP), had reported epidemiological associations with the risk of CAD (Additional file 1: Table S1). Details about the GWASs of these 37 traits are presented in Additional file 1: Table S2-S3.

### Calculation of heritability and genetic correlation

We applied LDSC to estimate the ${h}_{\mathrm{SNP}}^2$ of each trait using GWAS summary statistics [25]. We reported ${h}_{\mathrm{SNP}}^2$ on the liability scale for T2D and CAD, by assuming their population prevalence in East Asians and Europeans is 7.5% and 10.0% for T2D [26, 27], and 5.24% and 6.77% for CAD [28], respectively. In addition, we conducted cross-trait LDSC to quantify the genetic correlations (*r*_*g*_) between the 37 clinical factors and CAD, separately in each population [29]. We used the population-matched LD scores calculated from the 1000 Genomes Project (https://alkesgroup.broadinstitute.org/LDSCORE) [30]. The major histocompatibility complex region (chromosome 6: 25–34 Mb) was excluded due to its complex LD [31].

### Selection of IVs

Valid IVs need to be associated with the exposure (the relevance assumption), have no association with any confounders (the independence assumption), and have no association with the outcome conditional on the exposure (the exclusion restriction assumption) [8]. Assuming causal variants were largely shared between populations [32], we selected one set of IVs for both populations while using population-specific SNP effect sizes for the MR analyses in either population. We first meta-analyzed GWAS results from BBJ and UKB to identify potential causal variants for each clinical factor using the IVW fixed-effect model [22]. To meet the relevance assumption, we filtered SNPs with *P* ≥ 0.05 in either cohorts or opposite effects between cohorts, and then extracted independent and significant SNPs from the meta-analyzed results using the clumping algorithm in PLINK (v.1.90, LD *r*^2^ < 0.01, *P*_meta_ < 5×10^−8^, window size = 1Mb) [33]. The clumping step was performed twice based on East Asian and European reference panels from the 1000 Genomes Project, respectively [34]. The remaining SNPs were selected as candidate IVs for the clinical factor (exposure). The independence assumption is generally satisfied because of the random assortment of genetic alleles during meiosis, but it is challenging to ensure the exclusion restriction assumption due to the ubiquitous horizontal pleiotropic effects. Therefore, we adopted a stringent criterion to exclude candidate IVs in significant association (*P*_meta_ < 5×10^−8^) with any other clinical factors [8], with some exceptions detailed in Additional file 1: Table S4. Furthermore, we removed candidate IVs that failed the Mendelian randomization pleiotropy residual sum and outlier (MR-PRESSO) test (*P* < 0.05) [35]. The same IV selection procedure was applied to CAD in the reverse MR analyses.

### UVMR analyses

UVMR analyses were performed in East Asians and Europeans separately, followed by a fixed-effect meta-analysis [36]. We performed Cochran’s *Q* test to examine heterogeneity between populations. Four UVMR methods were applied: the MR-Corr method [37], the IVW method [38], the Bayesian weighted Mendelian randomization (BWMR) method [39], and the robust adjusted profile score (RAPS) method [40]. MR-Corr is designed to address the correlated horizontal pleiotropy issue [37]. We presented MR-Corr estimates as our main results. The IVW method combines effect estimates from individual IVs using a multiplicative random effect model to handle dispersion of effect estimates due to pleiotropy [38]. Both BWMR and RAPS can handle the measurement error and horizontal pleiotropy by adopting either a Bayesian weighting scheme [39] or a robust adjusted profile score [40]. We calculated the odds ratio (OR) and the corresponding 95% confidence interval (CI) of CAD per SD increment of a quantitative exposure or per unit change on the log odds scale of a binary exposure. SDs for quantitative traits were presented in Additional file 1: Table S2-S3, in which the values for UKB (SD_UKB_) were calculated using individual phenotype data of 472,671 white-British participants, and the values for BBJ (SD_BBJ_) were obtained from reference [16]. While SD_UKB_ ≈ SD_BBJ_ for most traits, we rescaled the SNP effect sizes (and standard errors) of IVs from BBJ by SD_UKB_/SD_BBJ_, such that the MR causal effect estimates from two populations are in the same unit of SD_UKB_. Bonferroni-corrected thresholds (0.05/37 = 0.00135 in the forward MR and 0.05/4 = 0.0125 in the reverse MR) were adopted to account for multiple testing. We performed Steiger’s directionality test based on all IVs to confirm the bi-directional causal relationships between hemoglobin (Hb) and CAD [41]. MR analyses were conducted using the MR.Corr2 [37], TwoSampleMR [42], BWMR [39], and mr.raps [40] R packages.

To evaluate the validity of UVMR analyses, we computed the proportion of variance explained by each IV (*PVE*) and the corresponding *F* statistic as $F=\frac{\mathrm{\it PVE}\times \left(N-2\right)}{1-\mathrm{\it PVE}}$, where *N* represents the effective GWAS sample size [43]. An IV with *F* > 10 was considered as a strong instrument [8]. For multiple IVs, we computed the mean *F* statistic across IVs [43]. We inspected the heterogeneity of MR estimates by Cochran’s *Q* test [44], and the potential directional horizontal pleiotropy by a funnel plot [44]. Besides, we estimated the potential bias introduced by sample overlap as *βr*/*F*, where *β* is plugged-in with the MR-Corr estimate and *r* is the overlapping rate of sample between the GWASs of the exposure and the outcome [43].

### MVMR analyses

We performed MVMR analyses using the MVMR-Robust method [45]. We first jointly analyzed 6 cardiometabolic exposures, including height, BMI, HbA1c, low-density lipoprotein cholesterol (LDL-C), triglyceride (TG), and systolic blood pressure (SBP) to estimate their independent causal effect on CAD after adjusting for each other. We then performed MVMR analyses for each of red blood cell count (RBC), Hb, hematocrit (Ht), and uric acid (UA), adjusting for the aforementioned 6 cardiometabolic factors. We did not adjust for T2D, fasting glucose (FG), total cholesterol (TC), and diastolic blood pressure (DBP) due to their high correlation with HbA1c, LDL-C, and SBP. For each MVMR, we took the union set of candidate IVs of all exposures, and then extracted independent (LD *r*^2^ < 0.01) IVs, preferentially keeping IVs of the risk factor of interest. We excluded candidate IVs associated (*P*_meta_ < 5×10^−8^) with any clinical factors not included as exposures to remove pleiotropy. We calculated the conditional *F* statistic to assess the instrumental strength with the phenotypic correlations obtained from UKB samples (Additional file 1: Fig. S2) [46].

## Results

### Heritability and genetic correlation

The SNP heritability ${h}_{\mathrm{SNP}}^2$ and pairwise genetic correlations of 37 clinical factors and CAD are displayed in Fig. 2. In Europeans, CAD was estimated to have ${h}_{\mathrm{SNP}}^2$ = 0.080 (standard error = 0.005), while the highest ${h}_{\mathrm{SNP}}^2$ was 0.417 (0.018) for height and the lowest was 0.022 (0.003) for basophil count (Baso). Heritability estimates were highly correlated between populations (Spearman’s *r*_s_ = 0.781, *P* = 7.21×10^−9^), but estimates based on UKB tended to be higher than those based on BBJ. Similarly, genetic correlations were largely consistent between populations (*r*_s_ = 0.758, *P* = 2.13×10^−132^), but many more significant correlations were identified in UKB due to larger sample sizes (Additional file 2: Table S5). In particular, CAD had a significant genetic correlation (*P* < 7.1×10^−5^, Bonferroni correction for 703 tests) with 22 out of 37 clinical factors in Europeans, indicating shared genetic architecture (Fig. 2B).

### Causal effects of clinical factors on CAD

**Fig. 3:**
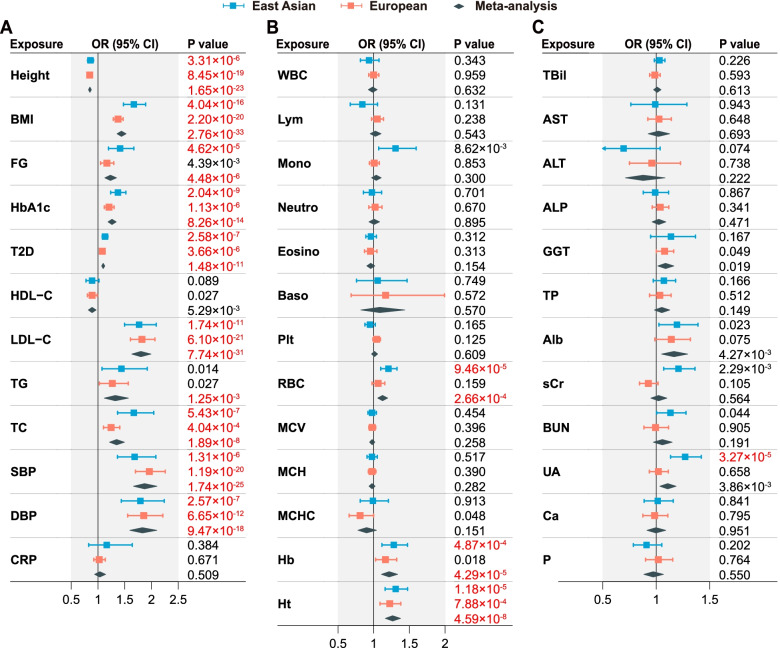
Causal effects of 37 clinical factors on CAD estimated by UVMR analyses. **A** Causal effects for 12 cardiometabolic risk factors. **B** Causal effects for 13 hematological indices. **C** Causal effects for 7 hepatic function biomarkers, 3 renal function biomarkers, and 2 serum electrolytes. Effect sizes are represented by OR per SD increment of a quantitative exposure or per unit change on the log odds scale of a binary exposure (T2D). The horizontal bars represent 95% CIs. Significant *P* values after Bonferroni correction (*P* < 0.05/37 = 0.00135) are highlighted in red

**Fig. 4:**
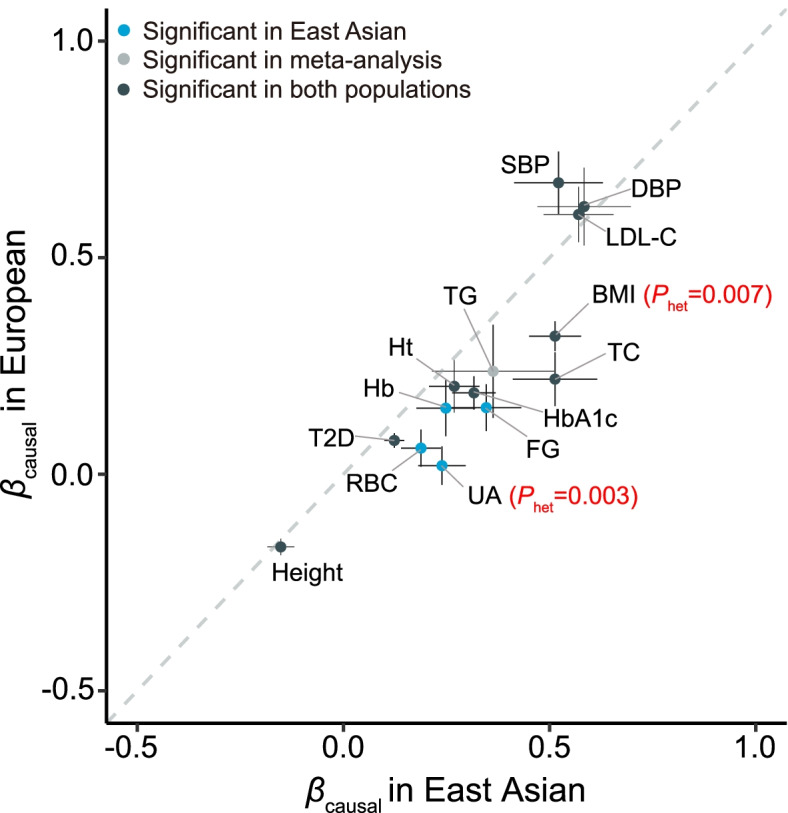
Comparison of causal effect sizes (*β*_causal_) on CAD for 14 significant risk factors. The *x*- and *y*-axes indicate estimates based on East Asian and European populations, respectively. The horizontal and vertical bars for each point indicate one standard error. *P*_het_ < 0.01 based on Cochran’s *Q* test of population heterogeneity are labeled in red

By UVMR analyses, we identified 14 significant risk factors: four in East Asians only, one in meta-analysis only, and nine in both populations (Table 1, Fig. 3, Additional file 1: Table S6-S8). The significant factors showed overall consistent effect estimates between populations (*r*_s_ = 0.947, *P* = 6.16×10^−9^), except for BMI (*P*_het_ = 0.007) and UA (*P*_het_ = 0.003) showing significant population heterogeneity (Fig. 4). The effect sizes in East Asians tended to be slightly larger than those in Europeans.

**Table 1:** UVMR analyses of 37 clinical factors on CAD

Category	Clinical factor (abbreviation)	SD^**a**^	OR (95% CI) per SD increment^b^		
			East Asian	European	Meta-analysis
Cardiometabolic	Height	9.3 cm	0.86 (0.81, 0.92)	0.85 (0.81, 0.88)	0.85 (0.82, 0.88)
	Body mass index (BMI)	4.8 kg/m^2^	1.67 (1.48, 1.89)	1.38 (1.29, 1.47)	1.44 (1.36, 1.53)
	Fasting glucose (FG)	21.82 mg/dL	1.41 (1.20, 1.67)	1.17 (1.05, 1.30)	1.23 (1.13, 1.35)
	Hemoglobin A1c (HbA1c)	0.60%	1.37 (1.24, 1.52)	1.21 (1.12, 1.30)	1.26 (1.19, 1.34)
	Type 2 diabetes (T2D)	-	1.13 (1.08, 1.19)	1.08 (1.05, 1.12)	1.10 (1.07, 1.13)
	High-density lipoprotein cholesterol (HDL-C)	14.80 mg/dL	0.89 (0.78, 1.02)	0.89 (0.80, 0.99)	0.89 (0.82, 0.97)
	Low-density lipoprotein cholesterol (LDL-C)	33.63 mg/dL	1.77 (1.50, 2.09)	1.82 (1.61, 2.06)	1.80 (1.63, 1.99)
	Triglyceride (TG)	90.68 mg/dL	1.44 (1.08, 1.92)	1.27 (1.03, 1.57)	1.32 (1.12, 1.57)
	Total cholesterol (TC)	44.21 mg/dL	1.67 (1.37, 2.04)	1.25 (1.10, 1.41)	1.35 (1.21, 1.50)
	Systolic blood pressure (SBP)	18.67 mmHg	1.68 (1.36, 2.08)	1.96 (1.70, 2.26)	1.87 (1.66, 2.10)
	Diastolic blood pressure (DBP)	10.14 mmHg	1.79 (1.44, 2.24)	1.85 (1.55, 2.21)	1.83 (1.59, 2.10)
	C-reactive protein (CRP)	0.44 mg/dL	1.16 (0.83, 1.64)	1.02 (0.92, 1.14)	1.03 (0.94, 1.14)
Hematological	White blood cell count (WBC)	2134.64/μL	0.94 (0.81, 1.07)	1.00 (0.93, 1.07)	0.98 (0.92, 1.05)
	Lymphocyte count (Lym)	1191.27/μL	0.84 (0.67, 1.05)	1.05 (0.97, 1.14)	1.02 (0.95, 1.10)
	Monocyte count (Mono)	276.57/μL	1.31 (1.07, 1.59)	1.01 (0.94, 1.08)	1.04 (0.97, 1.11)
	Neutrophil count (Neutro)	1416.78/μL	0.97 (0.86, 1.11)	1.02 (0.93, 1.12)	1.01 (0.93, 1.08)
	Eosinophil count (Eosino)	136.22/μL	0.96 (0.89, 1.04)	0.95 (0.87, 1.05)	0.96 (0.90, 1.02)
	Basophil count (Baso)	51.73/μL	1.06 (0.76, 1.47)	1.17 (0.68, 1.99)	1.08 (0.82, 1.44)
	Platelet count (Plt)	5.99×10^4^/μL	0.95 (0.88, 1.02)	1.04 (0.99, 1.09)	1.01 (0.97, 1.05)
	Red blood cell count (RBC)	40.94×10^4^/μL	1.21 (1.10, 1.33)	1.06 (0.98, 1.16)	1.12 (1.06, 1.20)
	Mean corpuscular volume (MCV)	4.41 fL	0.98 (0.92, 1.04)	0.98 (0.93, 1.03)	0.98 (0.94, 1.02)
	Mean corpuscular hemoglobin (MCH)	1.84 pg	0.98 (0.91, 1.05)	0.98 (0.92, 1.03)	0.98 (0.93, 1.02)
	Mean corpuscular hemoglobin concentration (MCHC)	1.07%	0.99 (0.81, 1.21)	0.81 (0.66, 1.00)	0.90 (0.78, 1.04)
	Hemoglobin (Hb)	1.23 g/dL	1.28 (1.11, 1.47)	1.16 (1.03, 1.32)	1.22 (1.11, 1.34)
	Hematocrit (Ht)	3.53%	1.31 (1.16, 1.48)	1.22 (1.09, 1.38)	1.27 (1.16, 1.38)
Hepatic function	Total bilirubin (TBil)	0.26 mg/dL	1.03 (0.98, 1.08)	0.99 (0.94, 1.04)	1.01 (0.97, 1.04)
	Aspartate aminotransferase (AST)	10.60 IU/L	0.99 (0.76, 1.29)	1.03 (0.92, 1.14)	1.02 (0.92, 1.13)
	Alanine aminotransferase (ALT)	14.16 IU/L	0.69 (0.47, 1.04)	0.96 (0.75, 1.23)	0.88 (0.71, 1.08)
	Alkaline phosphatase (ALP)	26.45 IU/L	0.99 (0.88, 1.12)	1.04 (0.96, 1.12)	1.02 (0.96, 1.09)
	*γ*-glutamyl transferase (GGT)	42.18 IU/L	1.14 (0.95, 1.37)	1.08 (1.00, 1.16)	1.09 (1.01, 1.17)
	Total protein (TP)	0.40 g/dL	1.07 (0.97, 1.18)	1.03 (0.94, 1.14)	1.05 (0.98, 1.13)
	Serum albumin (Alb)	0.26 g/dL	1.19 (1.02, 1.39)	1.14 (0.99, 1.32)	1.17 (1.05, 1.29)
Renal function	Serum creatinine (sCr)	0.20 mg/dL	1.21 (1.07, 1.36)	0.93 (0.84, 1.02)	1.02 (0.95, 1.10)
	Blood urea nitrogen (BUN)	3.90 mg/dL	1.13 (1.00, 1.28)	0.99 (0.88, 1.11)	1.06 (0.97, 1.15)
	Uric acid (UA)	1.35 mg/dL	1.27 (1.13, 1.42)	1.02 (0.93, 1.11)	1.11 (1.03, 1.19)
Electrolyte	Calcium (Ca)	0.38 mg/dL	1.01 (0.89, 1.16)	0.98 (0.87, 1.11)	1.00 (0.91, 1.09)
	Phosphorus (P)	0.50 mg/dL	0.91 (0.79, 1.05)	1.02 (0.90, 1.15)	0.97 (0.88, 1.07)

^a^ SD of each trait among white-British participants of UKB. OR of CAD was reported on the same SD unit of each quantitative trait for MR analyses in both Europeans and East Asians. ^b^ For T2D, OR of CAD was reported per unit change on the log odds scale

Ten out of 12 cardiometabolic risk factors showed consistent causal effects on CAD in both populations (Table 1, Fig. 3A, Additional file 1: Table S6). Height was protective with the meta-analysis odds ratio (OR_meta_) being 0.85 per 9.3 cm increment (95% CI: 0.82–0.88, *P* = 1.65×10^−23^). BMI presented stronger risk effect in East Asians (OR = 1.67 per 4.8 kg/m^2^ increment, 1.48–1.89, *P* = 4.04×10^−16^) than in Europeans (1.38, 1.29–1.47, *P* = 2.20×10^−20^, *P*_het_ = 0.007). Blood pressure and LDL-C showed the strongest risk effects, almost doubling the risk of CAD per SD increment (SBP: OR_meta_ = 1.87 per 18.67 mmHg increment, 1.66–2.10, *P* = 1.74×10^−25^; DBP: 1.83 per 10.14 mmHg increment, 1.59–2.10, *P* = 9.47×10^−18^; LDL-C: 1.80 per 33.63 mg/dL increment, 1.63–1.99, *P* = 7.74×10^−31^). In addition, we found significant risk effects of FG, HbA1c, T2D, TG, and TC, but not of high-density lipoprotein cholesterol (HDL-C) and C-reactive protein (CRP). Causal effect estimates from IVW, BWMR, and RAPS methods were consistent with estimates based on MR-Corr (Additional file 1: Table S6).

For 13 hematological indices, we found evidence of causal effects on CAD for RBC (OR = 1.21 per 40.94×10^4^/μL increment, 1.10–1.33, *P* = 9.46×10^−5^), Hb (1.28 per 1.23 g/dL increment, 1.11–1.47, *P* = 4.87×10^−4^), and Ht (1.31 per 3.53% increment, 1.16–1.48, *P* = 1.18×10^−5^) in East Asians, with similar results yielded by different MR methods (Tables 1 and 2, Fig. 3B, Additional file 1: Table S7). These causal effects were generally consistent between populations, despite relatively lower effect sizes in Europeans (OR = 1.06, 0.98–1.16, *P* = 0.159 for RBC; 1.16, 1.03–1.32, *P* = 0.018 for Hb; 1.22, 1.09–1.38, *P* = 7.88×10^−4^ for Ht). Reversely, we found no evidence of causal effect of CAD on RBC (*P*_meta_ = 0.051) or Ht (*P*_meta_ = 0.448, Table 2). In contrast, CAD had a significant causal effect on Hb (*P*_meta_ = 2.46×10^−3^), indicating bidirectional causal relationships, which were further supported by Steiger’s directionality test (Additional file 1: Table S9). No significant causal effects on CAD were found for other hematological indices, including counts of white blood cells (WBC), lymphocytes (Lym), monocytes (Mono), neutrophils (Neutro), eosinophils (Eosino), Baso, and platelets (Plt), as well as mean corpuscular volume (MCV), MCH, and mean corpuscular hemoglobin concentration (MCHC) (Table 1, Additional file 1: Table S7).

**Table 2:** Bi-directional UVMR analyses between RBC, Hb, Ht, UA and CAD

Exposure	Outcome	Population	IVs	***F***	MR-Corr		IVW		BWMR		RAPS	
					Effect size^**a**^	***P***	Effect size^**a**^	***P***	Effect size^**a**^	***P***	Effect size^**a**^	***P***
RBC	CAD	East Asian	136	11.24	1.21 (1.10, 1.33)	9.46×10^−5^	1.18 (1.07, 1.29)	1.02×10^−3^	1.20 (1.08, 1.33)	6.50×10^−4^	1.20 (1.09, 1.31)	1.44×10^−4^
		European	139	50.64	1.06 (0.98, 1.16)	0.159	1.06 (0.96, 1.17)	0.274	1.06 (0.96, 1.18)	0.270	1.02 (0.94, 1.12)	0.592
		Meta-analysis	-	-	1.12 (1.06, 1.20)	2.66×10^−4^	1.12 (1.04, 1.20)	1.67×10^−3^	1.13 (1.05, 1.21)	1.38×10^−3^	1.10 (1.03, 1.17)	2.87×10^−3^
Hb	CAD	East Asian	90	8.19	1.28 (1.11, 1.47)	4.87×10^−4^	1.21 (1.04, 1.40)	0.012	1.24 (1.05, 1.47)	0.013	1.25 (1.10, 1.42)	6.61×10^−4^
		European	89	40.78	1.16 (1.03, 1.32)	0.018	1.16 (1.00, 1.34)	0.058	1.17 (1.00, 1.36)	0.050	1.15 (1.01, 1.31)	0.033
		Meta-analysis	-	-	1.22 (1.11, 1.34)	4.29×10^−5^	1.18 (1.06, 1.31)	1.83×10^−3^	1.20 (1.07, 1.34)	1.80×10^−3^	1.20 (1.10, 1.31)	8.82×10^−5^
Ht	CAD	East Asian	105	8.66	1.31 (1.16, 1.48)	1.18×10^−5^	1.25 (1.09, 1.42)	9.62×10^−4^	1.30 (1.12, 1.50)	5.78×10^−4^	1.31 (1.16, 1.48)	1.20×10^−5^
		European	106	41.67	1.22 (1.09, 1.38)	7.88×10^−4^	1.22 (1.06, 1.41)	7.01×10^−3^	1.23 (1.06, 1.43)	6.48×10^−3^	1.19 (1.06, 1.34)	2.71×10^−3^
		Meta-analysis	-	-	1.27 (1.16, 1.38)	4.59×10^−8^	1.24 (1.12, 1.36)	2.07×10^−5^	1.26 (1.14, 1.40)	1.28×10^−5^	1.25 (1.15, 1.36)	2.14×10^−7^
UA	CAD	East Asian	57	26.02	1.27 (1.13, 1.42)	3.27×10^−5^	1.26 (1.11, 1.42)	1.81×10^−4^	1.26 (1.11, 1.43)	2.54×10^−4^	1.27 (1.13, 1.43)	3.77×10^−5^
		European	56	105.3	1.02 (0.93, 1.11)	0.658	1.02 (0.92, 1.13)	0.723	1.01 (0.91, 1.13)	0.789	1.01 (0.92, 1.11)	0.881
		Meta-analysis	-	-	1.11 (1.03, 1.19)	3.86×10^−3^	1.11 (1.03, 1.20)	6.61×10^−3^	1.11 (1.03, 1.21)	0.010	1.11 (1.03, 1.19)	6.49×10^−3^
CAD	RBC	East Asian	42	28.17	0.03 (0.00, 0.06)	0.099	0.02 (−0.01, 0.05)	0.108	0.03 (−0.01, 0.06)	0.110	0.03 (0.00, 0.05)	0.066
		European	44	30.74	0.01 (0.00, 0.03)	0.179	0.01 (−0.01, 0.03)	0.330	0.01 (−0.01, 0.04)	0.270	0.01 (−0.01, 0.02)	0.339
		Meta-analysis	-	-	0.01 (0.00, 0.03)	0.051	0.02 (0.00, 0.03)	0.081	0.02 (0.00, 0.04)	0.067	0.01 (0.00, 0.03)	0.079
CAD	Hb	East Asian	44	30.87	0.02 (0.00, 0.04)	0.075	0.02 (−0.01, 0.05)	0.124	0.02 (−0.01, 0.05)	0.120	0.02 (−0.01, 0.04)	0.119
		European	43	29.82	0.02 (0.00, 0.04)	0.014	0.02 (0.00, 0.04)	0.089	0.02 (0.00, 0.04)	0.101	0.02 (0.01, 0.04)	4.30×10^−3^
		Meta-analysis	-	-	0.02 (0.01, 0.03)	2.46×10^−3^	0.02 (0.00, 0.04)	0.022	0.02 (0.00, 0.04)	0.024	0.02 (0.01, 0.03)	1.15×10^−3^
CAD	Ht	East Asian	44	30.87	0.03 (0.00, 0.05)	0.061	0.03 (0.00, 0.05)	0.061	0.03 (0.00, 0.06)	0.055	0.02 (0.00, 0.05)	0.050
		European	41	30.37	0.00 (−0.02, 0.02)	0.723	0.00 (−0.03, 0.02)	0.769	0.00 (−0.03, 0.02)	0.766	0.00 (−0.02, 0.01)	0.559
		Meta-analysis	-	-	0.01 (−0.01, 0.02)	0.448	0.01 (−0.01, 0.03)	0.314	0.01 (−0.01, 0.03)	0.312	0.00 (−0.01, 0.02)	0.592
CAD	UA	East Asian	42	28.17	−0.01 (−0.03, 0.01)	0.431	−0.01 (−0.04, 0.02)	0.533	−0.01 (−0.04, 0.02)	0.585	0.00 (−0.03, 0.03)	0.914
		European	46	30.48	0.00 (−0.01, 0.02)	0.620	0.00 (−0.02, 0.02)	0.737	0.01 (−0.01, 0.02)	0.600	0.01 (−0.01, 0.02)	0.406
		Meta-analysis	-	-	0.00 (−0.01, 0.01)	0.972	0.00 (−0.02, 0.01)	0.944	0.00 (−0.01, 0.02)	0.888	0.00 (−0.01, 0.02)	0.507

^a^ Effect size corresponds to OR if the outcome is CAD or *β* if the outcome is RBC, Hb, Ht, or UA. 95% CI is indicated in the parentheses

Finally, we examined 7 hepatic function biomarkers, 3 renal function biomarkers, and 2 serum electrolytes (Tables 1 and 2, Fig. 3C, Additional file 1: Table S8). None of the 7 hepatic function biomarkers showed significant causal effects on CAD, including total bilirubin (TBil), aspartate aminotransferase (AST), alanine aminotransferase (ALT), alkaline phosphatase (ALP), *γ*-glutamyl transferase (GGT), TP, and serum albumin (Alb). Among renal function biomarkers, UA was found to increase the risk of CAD in East Asians (OR = 1.27 per 1.35 mg/dL increment, 95% CI: 1.13–1.42, *P* = 3.27×10^−5^), but not in Europeans (1.02, 0.93–1.11, *P* = 0.658, *P*_het_ = 0.003 between populations). Results based on different MR methods were similar and we found no causal role of CAD on UA (*P*_meta_ = 0.972, Table 2). In addition, we observed no evidence of a causal effect on CAD for serum creatinine (sCr) and blood urea nitrogen (BUN), as well as two serum electrolytes, calcium (Ca) and phosphorus (P).

We confirmed the validity of our UVMR analyses from three aspects. First, the mean *F* statistics for the valid IVs were all above 10, except for Lym, Mono, Hb, and Ht, which had a mean *F* slightly below 10 in BBJ, suggesting negligible concern on the weak instrumental bias (Table 2, Additional file 1: Table S6-S8) [8]. Second, although Cochran’s *Q* tests suggested heterogeneity in the causal estimates for some clinical factors (*P*_het_ < 0.01 in Additional file 1: Table S10), we observed no obvious directional horizontal pleiotropy in the funnel plots (Additional file 1: Fig. S3-S10). Furthermore, we confirmed that the sample overlap between GWASs of clinical factors and CAD in East Asians introduced little bias to our causal effect estimates (Additional file 1: Table S11).

### Causal effects independent of cardiometabolic factors

**Fig. 5:**
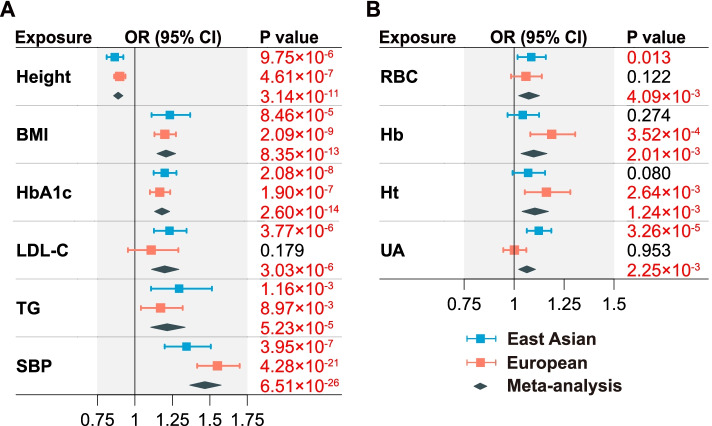
MVMR analyses of 10 significant clinical factors on CAD. **A** Independent effect estimates by joint analysis of six cardiometabolic factors. **B** Independent effect estimates for each of RBC, Hb, Ht, and UA after adjusting for six cardiometabolic factors in panel **A**. Effect sizes are represented by OR per SD increment in the exposure. The horizontal bars represent 95% CIs. *P* < 0.05 are highlighted in red

In the MVMR analysis including six cardiometabolic factors (Fig. 5A, Additional file 1: Table S12), all factors showed consistent and independent causal effects on CAD in East Asians and Europeans. SBP remained as the strongest risk factor with OR_meta_ = 1.47 (1.37–1.58, *P* = 6.51×10^−26^). We next examined the independent causal effects of RBC, Hb, Ht, and UA, conditioning on six cardiometabolic factors (Fig. 5B, Additional file 1: Table S12). Compared to the UVMR analyses, all four risk factors had attenuated effect sizes. RBC (OR_meta_ = 1.07, 1.02–1.13, *P* = 4.09×10^−3^), Hb (1.10, 1.03–1.16, *P* = 2.01×10^−3^), and Ht (1.10, 1.04–1.17, *P* = 1.24×10^−3^) had similar and significant causal effects, which may due to their strong genetic correlations (*r*_*g*_ ≥ 0.715, Additional file 2: Table S5). UA, on the other hand, reached significance in East Asians (OR = 1.12, 1.06–1.19, *P* = 3.26×10^−5^), but not in Europeans (1.00, 0.95–1.06, *P* = 0.953, *P*_het_ = 0.002). Except of height, the conditional *F* statistics of other clinical factors were lower than the conventional instrument strength threshold of 10, especially in the East Asian population (Additional file 1: Table S12). Nevertheless, the consistent causal effect estimates between MVMR and UVMR reduced concerns about false positive results due to potential weak instrumental bias.

## Discussion

Identification and comparison of causal risk factors in diverse populations can provide important information on developing prevention strategies for CAD. In this study, we evaluated the causality of 37 clinical factors on CAD by MR analyses in East Asians and Europeans. By analyzing large GWAS datasets under a unified MR framework, we identified 1 protective and 13 risk factors, the majority of which showed consistent effects between populations. These findings might inform prevention strategies and suggest potential therapeutic targets of CAD.

Our results highlight causal effects of red blood cell traits, including RBC, hemoglobin, and hematocrit, independent of traditional cardiometabolic factors. These three indices are highly correlated and all reflect the level of red cells in the blood. While red blood cells are traditionally viewed to function in oxygen transport, they are now recognized to actively participate in both arterial and venous thrombosis, as supported by clinical observations in patients with RBC abnormalities [47]. Furthermore, epidemiological studies have reported positive associations between hemoglobin concentration and both cardiovascular and all-cause mortality [48]. Potential mechanisms might involve an elevation of blood viscosity due to excess of RBC, vasoconstriction due to scavenging of nitric oxide by hemoglobin, promotion of platelet adhesion or activation by increasing hematocrit, or participation of normal RBC in thrombin generation [47]. By MR analysis, we confirmed the causal role of RBC on CAD, elevating the risk by nearly 10% per SD increase in RBC, hemoglobin, or hematocrit, even after adjusting for cardiometabolic factors. It is important to note that MR analysis based on GWAS summary statistics uses a linear approximation to estimate the causal effect, while the actual effect can be nonlinear, such as a U-shaped curve. In fact, both anemia and polycythemia vera have been reported to associate with increased cardiovascular risk [49, 50]. In particular, anemia can exacerbate cardiovascular complications, possibly due to compensatory consequences of hypoxia, including increased cardiac output and myocardial load, left ventricular hypertrophy, progressive heart enlargement, and atherosclerotic effects [49, 51]. Thus, our causal effect estimates should be interpreted with caution at extremes of red blood cell indices.

We also identified a potential causal role of UA on CAD, but the causal effects are heterogeneous between Europeans and East Asians. Consistent with the finding from Keenan et al. [52], we found no causal evidence of UA on CAD in Europeans, but we observed a significant causal effect in East Asians, which remained significant after adjusting for cardiometabolic risk factors. In fact, the pathogenetic role of UA on cardiovascular disease has been suggested by early experimental studies, potentially involving endothelial dysfunction, vascular smooth muscle cell proliferation, and inflammation [53]. The population heterogeneity of UA might be attributed to interaction with environmental factors, such as diet, because the prevalence of hyperuricemia and gout in East Asians is much higher than that in Europeans [54].

While 35 of the 37 clinical factors examined in our study have been reported to associate with CAD by epidemiological studies, we conclude no causal effects for 23 clinical factors, including HDL-C, CRP, hepatic function indices, white blood cell and platelet traits, most of the renal function indices, and two serum electrolytes. These negative results are as important as the positive discoveries for better understanding of the etiology of CAD. Many of the negative results are confirmatory to previous studies, including null associations for HDL-C [55], CRP [56], and Alb [57, 58], but some are contradictory. For example, Xu et al. [59] reported an increase in the liver function biomarker ALT could lower the risk of CAD in a MR analysis with two IVs. We found their results might be plagued by horizontal pleiotropy, because their IVs were significantly associated with TG in our meta-analysis (*P*_meta_ < 5×10^−8^). This example highlights a key strength of our study in selecting IVs with stringent criteria to exclude potential pleiotropy. In another example, Larsson et al. [60] reported a causal risk effect of serum calcium on CAD in an MR analysis with 6 IVs. While these SNPs were carefully selected to have no association with traditional cardiometabolic risk factors [60], we found 3 out of 6 SNPs were significantly associated with hepatic and renal function indices in our data (*P*_meta_ < 5×10^−8^), such that residual pleiotropy was possible. Furthermore, the sample size of the serum calcium GWAS used in Larsson et al. [60] (*n* = 61,079) was much smaller than those used in our analyses (*n* = 315,153 in UKB and 71,701 in BBJ). Our results are consistent with observational studies and RCTs that calcium intake from food or supplements has weak relationship with the risk of cardiovascular disease or all-cause mortality in the general population [61]. The effect of genetic predisposition to higher serum calcium levels on the risk of CAD needs future investigation.

Compared with previous MR studies, our study has several key strengths. First, our analyses are well powered by leveraging the largest publicly available GWAS datasets. Second, we have carefully selected IVs for each clinical factor, excluding potential horizontal pleiotropy with the other clinical factors to avoid false-positive findings, although our stringent criteria might be conservative by discarding SNPs with vertical pleiotropy. This issue is mitigated in MVMR analyses, in which SNPs associated with multiple exposures were included as the IVs. Third, our results are robust given consistent results derived from several different MR methods and two diverse ancestry groups. Fourth, our unified analysis framework facilitates direct comparison of causal effects among different clinical factors or between populations, leading to a more complete understanding of the etiology of CAD.

Nonetheless, there are several limitations of our study. First, the sample sizes of East Asian studies are still much smaller than those of Europeans. Thus, our meta-analysis results are likely dominated by European samples. Nevertheless, the Eurocentric bias in human genetics research is a well-recognized issue, and efforts have been made to promote research in non-European populations. Our study is among the first attempts to directly compare the causal effects of a large number of CAD risk factors across populations. Second, BBJ is a hospital-based patient-ascertained cohort, whereas UKB is a population-based healthy volunteer cohort. The cohort discrepancy may impair the comparability between populations, potentially explaining the slightly larger causal effect estimates in East Asians than in Europeans. In addition, there are concerns about the representativeness of BBJ and UKB to the general population [62, 63]. Nevertheless, it has been pointed out that a sufficiently large sample size with different levels of exposure is essential for the generalizability of associations between exposures and diseases [64, 65], and that the risk factor association results based on UKB are highly consistent to those from nationally representative cohorts [66]. Finally, our study is limited to 37 clinical factors with available summary statistics from large-scale GWAS, despite hundreds of CAD risk factors having been reported. We expect the aforementioned limitations to be resolved in future investigations with the increasing availability of data from large-scale population-based biobanks in many countries.

## Conclusions

We have identified 1 protective and 13 risk factors with reliable causal evidence on CAD, consistently in East Asians and Europeans. In addition to traditional cardiometabolic risk factors, red blood cells and uric acid showed significant independent risk effects. These findings have important implications for informing prevention strategies and potential therapeutic targets of CAD.

## Supplementary Information

**Additional file 1: Table S1**. Reported epidemiological associations between 37 clinical factors and CAD. **Table S2**. Characteristics of the subjects enrolled in the original GWASs of 37 clinical factors in BBJ. **Table S3**. Characteristics of the subjects enrolled in the original GWASs of 37 clinical factors in UKB. **Table S4**. Exceptions of potential pleiotropic clinical factors considered in the IV selection. **Table S6**. UVMR causal effect estimates of cardiometabolic factors on CAD based on IVW, BWMR and RAPS methods. **Table S7**. UVMR causal effect estimates of hematological indices on CAD based on IVW, BWMR and RAPS methods. **Table S8**. UVMR causal effect estimates of hepatic and renal function and serum electrolyte factors on CAD based on IVW, BWMR and RAPS methods. **Table S9**. Steiger’s test of directionality between Hb and CAD. **Table S10**. Heterogeneity of the causal estimates across IVs. **Table S11**. Potential bias in UVMR estimates due to sample overlap between GWAS of clinical factors and CAD based on BBJ. **Table S12**. MVMR causal effect estimates of 10 significant clinical factors on CAD after adjusting for 6 cardiometabolic factors. **Fig S1**. Selection procedure of 37 clinical factors for MR analyses. **Fig S2**. Phenotypic correlation between 10 significant clinical factors in the MVMR analyses. **Fig S3-S10**. Scatter plot and funnel plot for each exposure in the MR analyses in East Asians and Europeans.

**Additional file 2: Table S5**. Pairwise genetic correlation between the 37 selected clinical factors and CAD in East Asian and European populations.

**Additional file 3.** STROBE-MR-checklist.

## Data Availability

The GWAS summary data of 37 clinical factors were downloaded from BBJ (https://humandbs.biosciencedbc.jp/en/hum0014-v24) [16] and UKB (including T2D [19] and other 36 traits (http://www.nealelab.is/uk-biobank) [24]). The GWAS summary data of CAD were downloaded from BBJ (https://humandbs.biosciencedbc.jp/en/hum0014-v24) [17], CARDIoGRAMplusC4D (http://www.cardiogramplusc4d.org/) [20], and the FinnGen study (https://finngen.gitbook.io/documentation/) [21]. The UK Biobank individual-level data were obtained under the application number 63454. The Strobe MR checklist is provided in Additional file 3. Our analysis scripts are available at https://github.com/kaibios0101/MR-CAD [67].

## Abbreviations

Alb: Serum albumin
ALP: Alkaline phosphatase
ALT: Alanine aminotransferase
AST: Aspartate aminotransferase
Baso: Basophil count
BBJ: Biobank Japan
BMI: Body mass index
BUN: Blood urea nitrogen
BWMR: Bayesian weighted Mendelian randomization
Ca: Calcium
CAD: Coronary artery disease
CARDIoGRAMplusC4D: Coronary Artery Disease Genome-Wide Replication and Meta-analysis plus the Coronary Artery Disease Genetics
CI: Confidence interval
CRP: C-reactive protein
DBP: Diastolic blood pressure
Eosino: Eosinophil count
FG: Fasting glucose
GGT: *γ*-glutamyl transferase
GWAS: Genome-wide association studies
Hb: Hemoglobin
HbA1c: Hemoglobin A1c
HDL-C: High-density lipoprotein cholesterol
Ht: Hematocrit
IV: Instrumental variable
IVW: Inverse-variance-weighted
LDL-C: Low-density lipoprotein cholesterol
LDSC: Linkage-disequilibrium score regression
Lym: Lymphocyte count
MCH: Mean corpuscular hemoglobin
MCHC: Mean corpuscular hemoglobin concentration
MCV: Mean corpuscular volume
Mono: Monocyte count
MR: Mendelian randomization
MR-PRESSO: Mendelian randomization pleiotropy residual sum and outlier
MVMR: Multivariable MR
Neutro: Neutrophil count
P: Phosphorus
Plt: Platelet count
*PVE*: Proportion of variance explained by each IV
RAPS: Robust adjusted profile score
RBC: Red blood cell count
RCTs: Randomized controlled trials
SBP: Systolic blood pressure
sCr: Serum creatinine
SD: Standard deviation
SNP: Single nucleotide polymorphism
T2D: Type 2 diabetes
TBil: Total bilirubin
TC: Total cholesterol
TG: Triglyceride
TP: Total protein
UA: Uric acid
UKB: UK Biobank
UVMR: Univariable MR
WBC: White blood cell count
