# Supplementary material for: Mendelian randomization analysis of 37 clinical factors and coronary artery disease in East Asian and European populations
Source: Genome Med. 2022 Jun 14;14:63. doi: 10.1186/s13073-022-01067-1 (PMC9195360; doi:10.1186/s13073-022-01067-1)
Supplement: Supplementary file 1 — Additional file 1: Table S1. Reported epidemiological associations between 37 clinical factors and CAD. Table S2. Characteristics of the subjects enrolled in the original GWASs of 37 clinical factors in BBJ. Table S3. Characteristics of the subjects enrolled in the original GWASs of 37 clinical factors in UKB. Table S4. Exceptions of potential pleiotropic clinical factors considered in the IV selection. Table S6. UVMR causal effect estimates of cardiometabolic factors on CAD based on IVW, BWMR and RAPS methods. Table S7. UVMR causal effect estimates of hematological indices on CAD based on IVW, BWMR and RAPS methods. Table S8. UVMR causal effect estimates of hepatic and renal function and serum electrolyte factors on CAD based on IVW, BWMR and RAPS methods. Table S9. Steiger’s test of directionality between Hb and CAD. Table S10. Heterogeneity of the causal estimates across IVs. Table S11. Potential bias in UVMR estimates due to sample overlap between GWAS of clinical factors and CAD based on BBJ. Table S12. MVMR causal effect estimates of 10 significant clinical factors on CAD after adjusting for 6 cardiometabolic factors. Fig S1. Selection procedure of 37 clinical factors for MR analyses. Fig S2. Phenotypic correlation between 10 significant clinical factors in the MVMR analyses. Fig S3-S10. Scatter plot and funnel plot for each exposure in the MR analyses in East Asians and Europeans. [file 13073_2022_1067_MOESM1_ESM.docx]

**SUPPLEMENTARY MATERIAL**

**Mendelian randomization analysis of 37 clinical factors and coronary artery disease in East Asian and European populations**

Kai Wang^1^, Xian Shi^1^, Ziwei Zhu^1^, Xingjie Hao^1^, Liangkai Chen^2^, Shanshan Cheng^1^, Roger S. Y. Foo^3,4^ and Chaolong Wang^1,#^

^1^ Department of Epidemiology and Biostatistics, Ministry of Education Key Laboratory of Environment and Health, School of Public Health, Tongji Medical College, Huazhong University of Science and Technology, Wuhan, China

^2^ Department of Nutrition and Food Hygiene, Hubei Key Laboratory of Food Nutrition and Safety, School of Public Health, Tongji Medical College, Huazhong University of Science and Technology, Wuhan, China

^3^ Cardiovascular Research Institute, Centre for Translational Medicine, National University Health System, Singapore

^4^ Genome Institute of Singapore, Singapore

# Correspondence: [chaolong@hust.edu.cn](mailto:chaolong@hust.edu.cn) (Chaolong Wang)

**Abbreviations**

Alb, Serum albumin; ALP, Alkaline phosphatase; ALT, Alanine aminotransferase; AST, Aspartate aminotransferase; Baso, Basophil count; BBJ, Biobank Japan; BMI, Body mass index; BUN, Blood urea nitrogen; BWMR, Bayesian weighted Mendelian randomization; Ca, Calcium; CAD, Coronary artery disease; CI, Confidence interval; CRP, C-reactive protein; DBP, Diastolic blood pressure; Eosino, Eosinophil count; FG, Fasting glucose; GGT, *γ*-glutamyl transferase; GWAS, Genome-wide association studies; Hb, Hemoglobin; HbA1c, Hemoglobin A1c; HDL-C, High-density lipoprotein cholesterol; Ht, Hematocrit; IV, Instrumental variable; IVW, Inverse-variance-weighted; LDL-C, Low-density lipoprotein cholesterol; LDSC, Linkage-disequilibrium score regression; Lym, Lymphocyte count; MCH, Mean corpuscular hemoglobin; MCHC, Mean corpuscular hemoglobin concentration; MCV, Mean corpuscular volume; Mono, Monocyte count; MR, Mendelian randomization; MR-PRESSO, Mendelian randomization pleiotropy residual sum and outlier; MVMR, Multivariable MR; Neutro, Neutrophil count; P, Phosphorus; Plt, Platelet count; RAPS, Robust adjusted profile score; RBC, Red blood cell count; SBP, Systolic blood pressure; sCr, Serum creatinine; SD, Standard deviation; T2D, Type 2 diabetes; TBil, Total bilirubin; TC, Total cholesterol; TG, Triglyceride; TP, Total protein; UA, Uric acid; UKB, UK Biobank; UVMR, Univariable MR; WBC, White blood cell count.

**Table S1. Reported epidemiological** **associations between 37 clinical factors and CAD.**

| **Category** | **Clinical factor** | **Reference (Author, Year, PMID)** | **Study design** |
| --- | --- | --- | --- |
| Cardiometabolic | Height | Paajanen *et al*., 2010, 20530501 | Systematic review |
|  | BMI | Riaz *et al*., 2018, 30646365 | Systematic review |
|  | FG | Emerging Risk Factors Collaboration *et al*., 2010, 20609967 | Meta-analysis |
|  | HbA1c | Pai *et al*., 2013, 23537807 | Prospective study |
|  | T2D | Emerging Risk Factors Collaboration *et al*., 2010, 20609967 | Meta-analysis |
|  | HDL-C | Emerging Risk Factors Collaboration *et al*., 2009, 19903920 | Prospective study |
|  | LDL-C | Cholesterol Treatment Trialists' (CTT) Collaborators *et al*., 2012, 22607822 | Meta-analysis |
|  | TG | Emerging Risk Factors Collaboration *et al*., 2009, 19903920 | Prospective study |
|  | TC | Dugani *et al*., 2021, 33471027 | Prospective study |
|  | SBP | Hoogen*et al*., 2000, 10620642 | Prospective study |
|  | DBP | Hoogen*et al*., 2000, 10620642 | Prospective study |
|  | CRP | Wilson *et al*., 2006, 16337036 | Review |
| Hematological | WBC | Horne *et al*., 2005, 15893180 | Prospective study |
|  | Lym | Horne *et al*., 2005, 15893180 | Prospective study |
|  | Mono | Horne *et al*., 2005, 15893180 | Prospective study |
|  | Neutro | Horne *et al*., 2005, 15893180 | Prospective study |
|  | Eosino | Sweetnam *et al*., 1997, 9048515 | Prospective study |
|  | Baso | Groot *et al*., 2020, 31812706 | Prospective study |
|  | Plt | Nikolsky *et al*., 2006, 17437727 | Prospective study |
|  | RBC | Byrnes *et al*., 2017, 28811305 | Review |
|  | MCV | Myojo *et al*., 2012, 22222418 | Prospective study |
|  | MCH | - | - |
|  | MCHC | Huang *et al*., 2016, 27294086 | Prospective study |
|  | Hb | Chonchol *et al*., 2008, 18294483 | Prospective study |
|  | Ht | Brown *et al*., 2001, 11579356 | Prospective study |
| Hepatic function | TBil | Lai *et al*., 2012, 28879775 | Prospective study |
|  | AST | Ndrepepa *et al*., 2020, 32917497 | Prospective study |
|  | ALT | Schindhelm *et al*., 2006, 16832839 | Review |
|  | ALP | Tonelli*et al*., 2009, 19841303 | Prospective study |
|  | GGT | Ndrepepa *et al*., 2018, 29175647 | Review |
|  | TP | - | - |
|  | Alb | Djoussé *et al*., 2002, 12460872 | Prospective study |
| Renal function | sCr | Sibilitz *et al*., 2014, 25222342 | Prospective study |
|  | BUN | Kirtane *et al*., 2005, 15936606 | Prospective study |
|  | UA | Johnson *et al*., 2003, 12707287 | Review |
| Serum electrolyte | Ca | Chung *et al*., 2016, 27776363 | Systematic review |
|  | P | Onufrak *et al*., 2009, 18980959 | Prospective study |

We performed post-hoc literature search of the epidemiological associations between each clinical factor and CAD in PubMed. We only listed one representative study for each clinical factor. For MCH and TP, no reported epidemiological associations were found.

**Table S2. Characteristics of the subjects enrolled in the original GWASs of 37 clinical factors in BBJ.**

| **Category** | **Trait** | **Sample size^*^** | **Female (%)** | **Age (years, Mean±SD)** | **Mean±SD** | **Unit** | **Standardization** | **Reference**  **(PMID)** |
| --- | --- | --- | --- | --- | --- | --- | --- | --- |
| Cardiometabolic | Height | 159,195 | 45.8 | 63.3±13.2 | 165.4±6.6 | cm | Z-score | 31562340 |
|  | BMI | 158,284 | 45.7 | 62.6±14.0 | 23.3±3.7 | kg/m^2^ | Log transformation + Inverse-rank normalization | 28892062 |
|  | FG | 93,146 | 47.4 | 63.0±13.4 | 106.32±21.08 | mg/dL | Inverse-rank normalization | 29403010 |
|  | HbA1c | 42,970 | 41.2 | 64.8±11.5 | 5.49±0.60 | % | Inverse-rank normalization | 29403010 |
|  | T2D | 36,614/155,150 | 46.2 | 64.7±10.1 | - | - | - | 30718926 |
|  | HDL-C | 70,657 | 42.6 | 63.7±12.1 | 54.75±15.43 | mg/dL | Z-score | 29403010 |
|  | LDL-C | 72,866 | 42.8 | 63.9±12.0 | 130.81±40.97 | mg/dL | Z-score | 29403010 |
|  | TG | 105,597 | 44.1 | 63.6±12.5 | 133.17±71.86 | mg/dL | Log transformation + Z-score | 29403010 |
|  | TC | 128,305 | 45.0 | 63.1±12.9 | 199.17±38.56 | mg/dL | Z-score | 29403010 |
|  | SBP | 136,597 | 44.9 | 62.6±13.3 | 134.68±19.69 | mmHg | Z-score | 29403010 |
|  | DBP | 136,615 | 44.9 | 62.6±13.3 | 79.35±12.13 | mmHg | Z-score | 29403010 |
|  | CRP | 75,391 | 42.8 | 63.6±12.8 | 0.24±0.27 | mg/dL | Inverse-rank normalization | 29403010 |
| Hematological | WBC | 107,964 | 45.8 | 62.7±13.1 | 6126.44±1856.41 | /μL | Z-score | 29403010 |
|  | Lym | 62,076 | 46.6 | 62.3±13.1 | 1791.08±702.76 | /μL | Inverse-rank normalization | 29403010 |
|  | Mono | 62,076 | 46.6 | 62.3±13.1 | 376.19±170.94 | /μL | Inverse-rank normalization | 29403010 |
|  | Neutro | 62,076 | 46.6 | 62.3±13.1 | 3673.61±1501.73 | /μL | Inverse-rank normalization | 29403010 |
|  | Eosino | 62,076 | 46.6 | 62.3±13.1 | 189.13±191.08 | /μL | Inverse-rank normalization | 29403010 |
|  | Baso | 62,076 | 46.6 | 62.3±13.1 | 36.31±33.68 | /μL | Inverse-rank normalization | 29403010 |
|  | Plt | 108,208 | 45.8 | 62.7±13.2 | 22.63±6.78 | 10^4^/μL | Z-score | 29403010 |
|  | RBC | 108,794 | 45.8 | 62.7±13.2 | 434.93±55.21 | 10^4^/μL | Z-score | 29403010 |
|  | MCV | 108,256 | 45.7 | 62.7±13.2 | 92.88±5.19 | fL | Z-score | 29403010 |
|  | MCH | 108,054 | 45.7 | 62.7±13.2 | 31.04±2.01 | pg | Z-score | 29403010 |
|  | MCHC | 108,728 | 45.7 | 62.7±13.2 | 33.38±1.07 | % | Z-score | 29403010 |
|  | Hb | 108,769 | 45.8 | 62.7±13.2 | 13.45±1.74 | g/dL | Z-score | 29403010 |
|  | Ht | 108,757 | 45.8 | 62.7±13.2 | 40.27±4.82 | % | Z-score | 29403010 |
| Hepatic function | TBil | 110,207 | 45.7 | 63.0±13.2 | 0.62±0.29 | mg/dL | Inverse-rank normalization | 29403010 |
|  | AST | 134,154 | 45.7 | 63.0±13.3 | 22.96±7.79 | IU/L | Log transformation + Z-score | 29403010 |
|  | ALT | 134,182 | 45.8 | 63.2±13.2 | 22.33±11.94 | IU/L | Log transformation + Z-score | 29403010 |
|  | ALP | 105,030 | 44.9 | 63.1±13.2 | -^†^ | IU/L | Inverse-rank normalization | 29403010 |
|  | GGT | 118,309 | 46.9 | 63.1±13.2 | 35.00±25.39 | IU/L | Log transformation + Z-score | 29403010 |
|  | TP | 113,509 | 44.9 | 63.0±13.3 | 7.11±0.56 | g/dL | Inverse-rank normalization | 29403010 |
|  | Alb | 102,223 | 44.3 | 63.4±13.0 | 4.22±0.42 | g/dL | Inverse-rank normalization | 29403010 |
| Renal function | sCr | 142,097 | 45.2 | 62.9±13.2 | 0.77±0.22 | mg/dL | Log transformation + Z-score | 29403010 |
|  | BUN | 139,818 | 45.1 | 62.8±13.2 | 15.44±4.77 | mg/dL | Log transformation + Z-score | 29403010 |
|  | UA | 109,029 | 46.2 | 62.9±13.0 | 5.32±1.45 | mg/dL | Z-score | 29403010 |
| Serum electrolyte | Ca | 71,701 | 44.9 | 63.9±12.6 | 9.19±0.52 | mg/dL | Inverse-rank normalization | 29403010 |
|  | P | 42,793 | 42.7 | 63.8±12.7 | 3.35±0.61 | mg/dL | Inverse-rank normalization | 29403010 |

^*^ Sample size for T2D was presented as cases/controls.

^†^ The mean and SD of ALP reported by BBJ (234.20±83.13 IU/L) were far away from the normal range in the general population, likely due to mistakes.

**Table S3. Characteristics of the subjects enrolled in the original GWASs of 37 clinical factors in UKB.**

| **Category** | **Trait** | **Sample size^*^** | **Female** (**%**) | **Age (years, Mean±SD)** ^†^ | **Mean±SD** ^†^ | **Unit** | **Standardization** | **Reference**  **(PMID)** |
| --- | --- | --- | --- | --- | --- | --- | --- | --- |
| Cardiometabolic | Height | 360,388 | 54.0 | 56.8±8.0 | 168.6±9.3 | cm | Inverse-rank normalization | Neale Lab **^‡^** |
|  | BMI | 354,831 | 54.0 | 56.8±8.0 | 27.4±4.8 | kg/m^2^ | Inverse-rank normalization | Neale Lab **^‡^** |
|  | FG | 314,916 | 54.0 | 56.8±8.0 | 92.10±21.82 | mg/dL | Inverse-rank normalization | Neale Lab **^‡^** |
|  | HbA1c | 344,182 | 54.0 | 56.8±8.0 | 5.44±0.60 | % | Inverse-rank normalization | Neale Lab **^‡^** |
|  | T2D | 18,197/424,620 | 47.8 | 56.8±8.0 | - | - | - | 30297969 |
|  | HDL-C | 315,133 | 54.0 | 56.8±8.0 | 56.19±14.80 | mg/dL | Inverse-rank normalization | Neale Lab **^‡^** |
|  | LDL-C | 343,621 | 54.0 | 56.8±8.0 | 137.97±33.63 | mg/dL | Inverse-rank normalization | Neale Lab **^‡^** |
|  | TG | 343,992 | 54.0 | 56.8±8.0 | 155.09±90.68 | mg/dL | Inverse-rank normalization | Neale Lab **^‡^** |
|  | TC | 344,278 | 54.0 | 56.8±8.0 | 220.86±44.21 | mg/dL | Inverse-rank normalization | Neale Lab **^‡^** |
|  | SBP | 340,159 | 54.0 | 56.8±8.0 | 138.05±18.67 | mmHg | Inverse-rank normalization | Neale Lab **^‡^** |
|  | DBP | 340,162 | 54.0 | 56.8±8.0 | 82.22±10.14 | mmHg | Inverse-rank normalization | Neale Lab **^‡^** |
|  | CRP | 343,524 | 54.0 | 56.8±8.0 | 0.26±0.44 | mg/dL | Inverse-rank normalization | Neale Lab **^‡^** |
| Hematological | WBC | 350,470 | 54.0 | 56.8±8.0 | 6895.54±2134.64 | /μL | Inverse-rank normalization | Neale Lab **^‡^** |
|  | Lym | 349,856 | 54.0 | 56.8±8.0 | 1954.86±1191.27 | /μL | Inverse-rank normalization | Neale Lab **^‡^** |
|  | Mono | 349,856 | 54.0 | 56.8±8.0 | 478.24±276.57 | /μL | Inverse-rank normalization | Neale Lab **^‡^** |
|  | Neutro | 349,856 | 54.0 | 56.8±8.0 | 4248.31±1416.78 | /μL | Inverse-rank normalization | Neale Lab **^‡^** |
|  | Eosino | 349,856 | 54.0 | 56.8±8.0 | 173.81±136.22 | /μL | - | Neale Lab **^‡^** |
|  | Baso | 349,856 | 54.0 | 56.8±8.0 | 33.96±51.73 | /μL | - | Neale Lab **^‡^** |
|  | Plt | 350,474 | 54.0 | 56.8±8.0 | 25.33±5.99 | 10^4^/μL | Inverse-rank normalization | Neale Lab **^‡^** |
|  | RBC | 350,475 | 54.0 | 56.8±8.0 | 450.95±40.94 | 10^4^/μL | Inverse-rank normalization | Neale Lab **^‡^** |
|  | MCV | 350,473 | 54.0 | 56.8±8.0 | 91.32±4.41 | fL | Inverse-rank normalization | Neale Lab **^‡^** |
|  | MCH | 350,468 | 54.0 | 56.8±8.0 | 31.54±1.84 | pg | Inverse-rank normalization | Neale Lab **^‡^** |
|  | MCHC | 350,472 | 54.0 | 56.8±8.0 | 34.54±1.07 | % | Inverse-rank normalization | Neale Lab **^‡^** |
|  | Hb | 350,474 | 54.0 | 56.8±8.0 | 14.19±1.23 | g/dL | Inverse-rank normalization | Neale Lab **^‡^** |
|  | Ht | 350,475 | 54.0 | 56.8±8.0 | 41.11±3.53 | % | Inverse-rank normalization | Neale Lab **^‡^** |
| Hepatic function | TBil | 342,829 | 54.0 | 56.8±8.0 | 0.53±0.26 | mg/dL | Inverse-rank normalization | Neale Lab **^‡^** |
|  | AST | 342,990 | 54.0 | 56.8±8.0 | 26.21±10.60 | IU/L | Inverse-rank normalization | Neale Lab **^‡^** |
|  | ALT | 344,136 | 54.0 | 56.8±8.0 | 23.54±14.16 | IU/L | Inverse-rank normalization | Neale Lab **^‡^** |
|  | ALP | 344,292 | 54.0 | 56.8±8.0 | 83.58±26.45 | IU/L | Inverse-rank normalization | Neale Lab **^‡^** |
|  | GGT | 344,104 | 54.0 | 56.8±8.0 | 37.33±42.18 | IU/L | Inverse-rank normalization | Neale Lab **^‡^** |
|  | TP | 314,921 | 54.0 | 56.8±8.0 | 7.24±0.40 | g/dL | Inverse-rank normalization | Neale Lab **^‡^** |
|  | Alb | 315,268 | 54.0 | 56.8±8.0 | 4.52±0.26 | g/dL | Inverse-rank normalization | Neale Lab **^‡^** |
| Renal function | sCr | 344,104 | 54.0 | 56.8±8.0 | 0.82±0.20 | mg/dL | Inverse-rank normalization | Neale Lab **^‡^** |
|  | BUN | 344,052 | 54.0 | 56.8±8.0 | 15.20±3.90 | mg/dL | Inverse-rank normalization | Neale Lab **^‡^** |
|  | UA | 343,836 | 54.0 | 56.8±8.0 | 5.20±1.35 | mg/dL | Inverse-rank normalization | Neale Lab **^‡^** |
| Serum electrolyte | Ca | 315,153 | 54.0 | 56.8±8.0 | 9.52±0.38 | mg/dL | Inverse-rank normalization | Neale Lab **^‡^** |
|  | P | 314,658 | 54.0 | 56.8±8.0 | 3.59±0.50 | mg/dL | Inverse-rank normalization | Neale Lab **^‡^** |

^*^ Sample size for T2D was presented as cases/controls.

^†^ Based on 472,671 white-British participants in UKB.

**^‡^** GWAS summary statistics were downloaded from http://www.nealelab.is/uk-biobank.

**Table S4. Exceptions of potential pleiotropic clinical factors considered in the IV selection.**

| **Exposure** | **Exceptions** | **Reason** |
| --- | --- | --- |
| FG | HbA1c, T2D | These are highly correlated glycemic traits, and T2D is often diagnosed based on FG and HbA1c. |
| HbA1c | FG, T2D |  |
| T2D | FG, HbA1c |  |
| HDL-C / LDL-C / TG | TC | TC includes HDL-C, LDL-C, and TG. |
| TC | HDL-C, LDL-C, and TG |  |
| Lym / Mono / Neutro / Eosino / Baso | WBC | WBC includes Lym, Mono, Neutro, Eosino, and Baso. |
| WBC | Lym / Mono / Neutro / Eosino / Baso |  |
| RBC | MCV, MCHC, MCH, Hb, Ht | These are closely related red blood cell traits. |
| MCV | RBC, MCHC, MCH, Hb, Ht |  |
| MCHC | RBC, MCV, MCH, Hb, Ht |  |
| MCH | RBC, MCV, MCHC, Hb, Ht |  |
| Hb | RBC, MCV, MCHC, MCH, Ht |  |
| Ht | RBC, MCV, MCHC, MCH, Hb |  |
| Alb | TP | TP includes Alb. |
| TP | Alb |  |

To minimize potential pleiotropic effects, for each exposure, we remove candidate IVs in significant association (*P*_meta_ < 5×10^-8^) with any of the other 36 clinical factors with exceptions listed in the table.

**Table S5. Pairwise genetic correlation between the 37 selected clinical factors and CAD in East Asian and European populations.** *r*_g_, genetic correlation coefficient; SE, standard error. This table is provided in Additional file 2.

**Table S6. UVMR causal effect estimates of cardiometabolic factors on CAD based on IVW, BWMR and RAPS methods.**

| **Clinical factor** | **Population** | **IVs** | ***F*** | **IVW** | | **BWMR** | | **RAPS** | |
| --- | --- | --- | --- | --- | --- | --- | --- | --- | --- |
|  |  |  |  | ***OR* (95% CI)** | ***P*** | ***OR* (95% CI)** | ***P*** | ***OR* (95% CI)** | ***P*** |
| Height | East Asian | 857 | 25.26 | 0.86 (0.81, 0.92) | 1.50×10^-5^ | 0.86 (0.80, 0.92) | 1.73×10^-5^ | 0.86 (0.81, 0.92) | 2.73×10^-6^ |
|  | European | 888 | 62.35 | 0.85 (0.81, 0.89) | 1.05×10^-12^ | 0.85 (0.81, 0.88) | 8.03×10^-13^ | 0.85 (0.81, 0.88) | 5.38×10^-18^ |
| BMI | East Asian | 287 | 12.61 | 1.58 (1.38, 1.81) | 2.76×10^-11^ | 1.65 (1.42, 1.91) | 4.06×10^-11^ | 1.58 (1.39, 1.78) | 4.58×10^-13^ |
|  | European | 290 | 31.55 | 1.36 (1.27, 1.46) | 1.40×10^-17^ | 1.38 (1.28, 1.48) | 1.06×10^-17^ | 1.38 (1.29, 1.47) | 1.02×10^-20^ |
| FG | East Asian | 36 | 20.25 | 1.38 (1.16, 1.65) | 2.94×10^-4^ | 1.41 (1.17, 1.69) | 3.03×10^-4^ | 1.42 (1.20, 1.68) | 3.87×10^-5^ |
|  | European | 35 | 83.86 | 1.16 (1.02, 1.32) | 0.020 | 1.16 (1.02, 1.31) | 0.026 | 1.14 (1.02, 1.27) | 0.024 |
| HbA1c | East Asian | 72 | 12.70 | 1.32 (1.18, 1.48) | 2.62×10^-6^ | 1.35 (1.19, 1.54) | 2.81×10^-6^ | 1.38 (1.25, 1.52) | 3.08×10^-10^ |
|  | European | 70 | 105.25 | 1.20 (1.11, 1.31) | 1.94×10^-5^ | 1.20 (1.10, 1.31) | 3.06×10^-5^ | 1.21 (1.12, 1.30) | 1.00×10^-6^ |
| T2D | East Asian | 63 | 36.17 | 1.13 (1.07, 1.18) | 9.97×10^-7^ | 1.13 (1.08, 1.19) | 9.99×10^-7^ | 1.14 (1.08, 1.19) | 8.61×10^-8^ |
|  | European | 67 | 28.05 | 1.08 (1.04, 1.12) | 7.98×10^-5^ | 1.08 (1.04, 1.12) | 1.30×10^-4^ | 1.07 (1.04, 1.11) | 2.07×10^-5^ |
| HDL-C | East Asian | 53 | 15.15 | 0.89 (0.79, 1.01) | 0.072 | 0.89 (0.78, 1.01) | 0.071 | 0.89 (0.78, 1.02) | 0.097 |
|  | European | 55 | 68.76 | 0.89 (0.78, 1.01) | 0.070 | 0.88 (0.78, 1.00) | 0.045 | 0.93 (0.83, 1.03) | 0.157 |
| LDL-C | East Asian | 29 | 15.66 | 1.65 (1.36, 2.00) | 4.33×10^-7^ | 1.71 (1.39, 2.10) | 3.26×10^-7^ | 1.82 (1.55, 2.14) | 1.67×10^-13^ |
|  | European | 30 | 86.22 | 1.80 (1.51, 2.15) | 5.65×10^-11^ | 1.82 (1.52, 2.17) | 3.10×10^-11^ | 1.82 (1.61, 2.06) | 7.58×10^-21^ |
| TG | East Asian | 21 | 12.79 | 1.38 (0.88, 2.17) | 0.166 | 1.38 (0.86, 2.23) | 0.186 | 1.50 (1.04, 2.16) | 0.030 |
|  | European | 25 | 30.86 | 1.24 (0.94, 1.64) | 0.134 | 1.26 (0.94, 1.69) | 0.128 | 1.32 (1.05, 1.67) | 0.020 |
| TC | East Asian | 35 | 24.31 | 1.61 (1.22, 2.13) | 8.65×10^-4^ | 1.63 (1.21, 2.19) | 1.30×10^-3^ | 1.65 (1.36, 2.01) | 5.07×10^-7^ |
|  | European | 32 | 76.47 | 1.24 (1.06, 1.44) | 6.49×10^-3^ | 1.24 (1.06, 1.45) | 7.00×10^-3^ | 1.27 (1.12, 1.44) | 2.21×10^-4^ |
| SBP | East Asian | 61 | 10.25 | 1.56 (1.24, 1.96) | 1.57×10^-4^ | 1.65 (1.30, 2.11) | 4.35×10^-5^ | 1.75 (1.41, 2.16) | 2.83×10^-7^ |
|  | European | 60 | 34.73 | 1.91 (1.59, 2.29) | 4.11×10^-12^ | 1.96 (1.63, 2.37) | 1.91×10^-12^ | 1.90 (1.63, 2.21) | 1.74×10^-16^ |
| DBP | East Asian | 41 | 10.31 | 1.66 (1.29, 2.12) | 6.98×10^-5^ | 1.76 (1.33, 2.33) | 8.89×10^-5^ | 1.85 (1.47, 2.32) | 1.55×10^-7^ |
|  | European | 39 | 33.59 | 1.80 (1.42, 2.28) | 1.35×10^-6^ | 1.87 (1.47, 2.39) | 5.41×10^-7^ | 1.83 (1.54, 2.19) | 2.09×10^-11^ |
| CRP | East Asian | 27 | 12.63 | 1.14 (0.77, 1.69) | 0.526 | 1.14 (0.75, 1.74) | 0.542 | 1.12 (0.79, 1.58) | 0.515 |
|  | European | 26 | 138.36 | 1.02 (0.91, 1.14) | 0.711 | 1.03 (0.91, 1.16) | 0.656 | 1.03 (0.93, 1.14) | 0.621 |

Results derived from the MR-Corr method were presented in the main text.

**Table S7. UVMR causal effect estimates of hematological indices on CAD based on IVW, BWMR and RAPS methods.**

| **Clinical factor** | **Population** | **IVs** | ***F*** | **IVW** | | **BWMR** | | **RAPS** | |
| --- | --- | --- | --- | --- | --- | --- | --- | --- | --- |
|  |  |  |  | ***OR* (95% CI)** | ***P*** | ***OR* (95% CI)** | ***P*** | ***OR* (95% CI)** | ***P*** |
| WBC | East Asian | 136 | 11.81 | 0.94 (0.81, 1.09) | 0.429 | 0.94 (0.80, 1.11) | 0.449 | 0.96 (0.83, 1.10) | 0.547 |
|  | European | 138 | 53.82 | 1.00 (0.92, 1.09) | 0.959 | 1.00 (0.92, 1.09) | 0.961 | 0.98 (0.92, 1.06) | 0.679 |
| Lym | East Asian | 97 | 8.65 | 0.87 (0.68, 1.12) | 0.279 | 0.84 (0.63, 1.11) | 0.215 | 0.82 (0.67, 1.02) | 0.070 |
|  | European | 97 | 59.45 | 1.05 (0.95, 1.16) | 0.368 | 1.04 (0.94, 1.16) | 0.405 | 1.08 (0.99, 1.17) | 0.079 |
| Mono | East Asian | 93 | 9.36 | 1.29 (1.08, 1.54) | 5.72×10^-3^ | 1.31 (1.07, 1.61) | 8.66×10^-3^ | 1.34 (1.09, 1.65) | 6.14×10^-3^ |
|  | European | 99 | 83.12 | 1.01 (0.93, 1.09) | 0.896 | 1.01 (0.93, 1.09) | 0.839 | 1.00 (0.93, 1.07) | 0.931 |
| Neutro | East Asian | 73 | 10.29 | 0.99 (0.85, 1.16) | 0.924 | 0.99 (0.83, 1.17) | 0.881 | 1.00 (0.89, 1.13) | 0.975 |
|  | European | 73 | 58.14 | 1.02 (0.92, 1.13) | 0.730 | 1.02 (0.92, 1.13) | 0.735 | 1.02 (0.92, 1.12) | 0.740 |
| Eosino | East Asian | 103 | 11.37 | 0.96 (0.89, 1.05) | 0.383 | 0.96 (0.88, 1.05) | 0.389 | 0.96 (0.89, 1.03) | 0.240 |
|  | European | 104 | 83.11 | 0.96 (0.87, 1.06) | 0.386 | 0.96 (0.87, 1.06) | 0.440 | 0.98 (0.89, 1.07) | 0.591 |
| Baso | East Asian | 7 | 37.73 | 1.05 (0.72, 1.55) | 0.792 | 1.06 (0.71, 1.59) | 0.778 | 1.12 (0.81, 1.56) | 0.477 |
|  | European | 7 | 20.28 | 1.17 (0.67, 2.02) | 0.586 | 1.17 (0.66, 2.08) | 0.599 | 1.17 (0.64, 2.14) | 0.600 |
| Plt | East Asian | 207 | 14.09 | 0.95 (0.87, 1.04) | 0.293 | 0.95 (0.86, 1.04) | 0.272 | 0.94 (0.87, 1.02) | 0.124 |
|  | European | 208 | 72.05 | 1.04 (0.98, 1.11) | 0.188 | 1.04 (0.98, 1.11) | 0.163 | 1.04 (0.99, 1.10) | 0.109 |
| MCV | East Asian | 189 | 22.21 | 0.98 (0.91, 1.06) | 0.570 | 0.98 (0.91, 1.06) | 0.597 | 0.97 (0.91, 1.03) | 0.265 |
|  | European | 192 | 78.02 | 0.98 (0.93, 1.04) | 0.489 | 0.98 (0.92, 1.04) | 0.486 | 0.99 (0.94, 1.04) | 0.662 |
| MCH | East Asian | 148 | 23.83 | 0.97 (0.89, 1.07) | 0.564 | 0.97 (0.88, 1.06) | 0.504 | 0.97 (0.91, 1.04) | 0.443 |
|  | European | 150 | 86.86 | 0.98 (0.92, 1.03) | 0.401 | 0.97 (0.92, 1.03) | 0.348 | 0.98 (0.93, 1.04) | 0.570 |
| MCHC | East Asian | 20 | 28.67 | 0.99 (0.81, 1.21) | 0.940 | 0.99 (0.81, 1.20) | 0.905 | 1.01 (0.83, 1.23) | 0.897 |
|  | European | 20 | 37.79 | 0.81 (0.62, 1.05) | 0.108 | 0.78 (0.59, 1.03) | 0.083 | 0.76 (0.60, 0.95) | 0.016 |

Results derived from the MR-Corr method were presented in the main text, and results of RBC, Hb, and Ht were presented in Table 2.

**Table S8. UVMR causal effect estimates of hepatic and renal function and serum electrolyte factors on CAD based on IVW, BWMR and RAPS methods.**

| **Clinical factor** | **Population** | **IVs** | ***F*** | **IVW** | | **BWMR** | | **RAPS** | |
| --- | --- | --- | --- | --- | --- | --- | --- | --- | --- |
|  |  |  |  | ***OR* (95% CI)** | ***P*** | ***OR* (95% CI)** | ***P*** | ***OR* (95% CI)** | ***P*** |
| TBil | East Asian | 21 | 310.36 | 1.03 (0.98, 1.08) | 0.241 | 1.03 (0.98, 1.08) | 0.243 | 1.03 (0.98, 1.08) | 0.243 |
|  | European | 20 | 520.55 | 0.98 (0.94, 1.03) | 0.524 | 0.99 (0.94, 1.04) | 0.554 | 0.98 (0.94, 1.04) | 0.547 |
| AST | East Asian | 70 | 11.68 | 1.00 (0.78, 1.29) | 0.983 | 0.99 (0.75, 1.31) | 0.946 | 0.97 (0.74, 1.26) | 0.812 |
|  | European | 69 | 52.14 | 1.02 (0.89, 1.17) | 0.752 | 1.03 (0.90, 1.18) | 0.626 | 1.02 (0.92, 1.13) | 0.739 |
| ALT | East Asian | 22 | 11.63 | 0.73 (0.45, 1.18) | 0.196 | 0.75 (0.45, 1.26) | 0.282 | 0.83 (0.57, 1.23) | 0.361 |
|  | European | 22 | 30.25 | 0.95 (0.72, 1.26) | 0.746 | 0.96 (0.73, 1.28) | 0.798 | 0.92 (0.71, 1.18) | 0.509 |
| ALP | East Asian | 81 | 18.43 | 0.99 (0.87, 1.12) | 0.837 | 0.99 (0.87, 1.13) | 0.880 | 0.99 (0.88, 1.11) | 0.858 |
|  | European | 83 | 87.36 | 1.04 (0.95, 1.13) | 0.446 | 1.03 (0.94, 1.13) | 0.487 | 1.02 (0.94, 1.10) | 0.636 |
| GGT | East Asian | 92 | 22.43 | 1.13 (0.93, 1.38) | 0.230 | 1.13 (0.92, 1.39) | 0.253 | 1.15 (0.96, 1.38) | 0.119 |
|  | European | 94 | 84.22 | 1.08 (0.99, 1.18) | 0.078 | 1.09 (1.00, 1.18) | 0.060 | 1.09 (1.01, 1.17) | 0.026 |
| TP | East Asian | 84 | 15.58 | 1.06 (0.95, 1.19) | 0.266 | 1.07 (0.96, 1.20) | 0.236 | 1.05 (0.96, 1.16) | 0.265 |
|  | European | 86 | 39.02 | 1.03 (0.92, 1.15) | 0.600 | 1.02 (0.91, 1.15) | 0.707 | 1.04 (0.94, 1.15) | 0.495 |
| Alb | East Asian | 39 | 10.11 | 1.16 (0.98, 1.38) | 0.081 | 1.19 (0.99, 1.44) | 0.069 | 1.26 (1.09, 1.46) | 1.69×10^-3^ |
|  | European | 40 | 39.95 | 1.14 (0.96, 1.35) | 0.134 | 1.15 (0.96, 1.36) | 0.125 | 1.13 (0.97, 1.31) | 0.106 |
| sCr | East Asian | 141 | 12.42 | 1.19 (1.05, 1.35) | 6.74×10^-3^ | 1.21 (1.05, 1.39) | 7.29×10^-3^ | 1.23 (1.09, 1.39) | 9.97×10^-4^ |
|  | European | 141 | 48.75 | 0.93 (0.84, 1.03) | 0.171 | 0.94 (0.84, 1.04) | 0.218 | 0.95 (0.86, 1.04) | 0.273 |
| BUN | East Asian | 53 | 26.88 | 1.13 (0.98, 1.29) | 0.094 | 1.13 (0.98, 1.31) | 0.104 | 1.13 (1.00, 1.27) | 0.051 |
|  | European | 52 | 60.29 | 0.99 (0.87, 1.14) | 0.932 | 0.98 (0.85, 1.13) | 0.782 | 0.98 (0.88, 1.10) | 0.758 |
| Ca | East Asian | 44 | 10.24 | 1.01 (0.88, 1.17) | 0.880 | 1.01 (0.87, 1.18) | 0.861 | 0.97 (0.85, 1.11) | 0.678 |
|  | European | 46 | 54.00 | 0.98 (0.87, 1.11) | 0.790 | 0.98 (0.87, 1.11) | 0.793 | 1.00 (0.89, 1.12) | 0.946 |
| P | East Asian | 28 | 10.14 | 0.92 (0.81, 1.06) | 0.242 | 0.91 (0.78, 1.05) | 0.207 | 0.91 (0.78, 1.06) | 0.207 |
|  | European | 28 | 75.69 | 1.02 (0.86, 1.20) | 0.835 | 1.01 (0.86, 1.19) | 0.902 | 1.02 (0.90, 1.16) | 0.715 |

Results derived from the MR-Corr method were presented in the main text, and results of UA were presented in Table 2.

**Table S9. Steiger’s test of directionality between Hb and CAD.**

| **Exposure** | **Outcome** | **Population** | ***R*^2^**  **(Exposure)** | ***R*^2^**  **(Outcome)** | **Correct**  **causal direction** | ***P*_Steiger_** |
| --- | --- | --- | --- | --- | --- | --- |
| Hb | CAD | East Asian | 0.006769 | 0.001130 | TRUE | 3.38×10^-39^ |
| Hb | CAD | European | 0.010626 | 0.000695 | TRUE | 3.19×10^-244^ |
| CAD | Hb | East Asian | 0.014267 | 0.000544 | TRUE | 2.57×10^-148^ |
| CAD | Hb | European | 0.009724 | 0.000334 | TRUE | 2.82×10^-267^ |

**Table S10. Heterogeneity of the causal estimates across IVs.**

| **Clinical factor** | **East Asian** | | |  | **European** | | |
| --- | --- | --- | --- | --- | --- | --- | --- |
|  | **Cochran’s *Q*** | ***df*^*^** | ***P*** |  | **Cochran’s *Q*** | ***df*^*^** | ***P*** |
| Height | 1151.7 | 856 | 4.76×10^-11^ |  | 1341.9 | 887 | 3.34×10^-21^ |
| BMI | 416.7 | 286 | 6.90×10^-7^ |  | 356.2 | 289 | 4.24×10^-3^ |
| FG | 45.6 | 35 | 0.108 |  | 47.1 | 34 | 0.067 |
| HbA1c | 119.3 | 71 | 2.92×10^-4^ |  | 92.9 | 69 | 0.029 |
| T2D | 73.2 | 62 | 0.155 |  | 93.1 | 66 | 0.016 |
| HDL-C | 56.7 | 52 | 0.303 |  | 85.5 | 54 | 4.02×10^-3^ |
| LDL-C | 56.0 | 28 | 1.27×10^-3^ |  | 63.3 | 29 | 2.37×10^-4^ |
| TG | 36.6 | 20 | 0.013 |  | 38.2 | 24 | 0.033 |
| TC | 77.5 | 34 | 2.99×10^-5^ |  | 48.2 | 31 | 0.025 |
| SBP | 91.1 | 60 | 5.86×10^-3^ |  | 96.2 | 59 | 1.60×10^-3^ |
| DBP | 63.7 | 40 | 0.010 |  | 77.0 | 38 | 1.84×10^-4^ |
| CRP | 40.5 | 26 | 0.035 |  | 33.7 | 25 | 0.115 |
| WBC | 184.9 | 135 | 2.81×10^-3^ |  | 207.8 | 137 | 8.99×10^-5^ |
| Lym | 160.7 | 96 | 3.92×10^-5^ |  | 161.6 | 96 | 3.23×10^-5^ |
| Mono | 86.3 | 92 | 0.648 |  | 140.7 | 98 | 3.06×10^-3^ |
| Neutro | 133.7 | 72 | 1.38×10^-5^ |  | 92.4 | 72 | 0.053 |
| Eosino | 146.0 | 102 | 2.83×10^-3^ |  | 131.0 | 103 | 0.033 |
| Baso | 9.4 | 6 | 0.154 |  | 3.4 | 6 | 0.753 |
| Plt | 329.8 | 206 | 9.30×10^-8^ |  | 309.5 | 207 | 4.95×10^-6^ |
| RBC | 183.2 | 135 | 3.67×10^-3^ |  | 215.7 | 138 | 2.58×10^-5^ |
| MCV | 329.0 | 188 | 9.01×10^-10^ |  | 254.2 | 191 | 1.52×10^-3^ |
| MCH | 263.7 | 147 | 1.16×10^-8^ |  | 180.5 | 149 | 0.040 |
| MCHC | 22.4 | 19 | 0.267 |  | 28.3 | 19 | 0.078 |
| Hb | 149.4 | 89 | 6.37×10^-5^ |  | 129.4 | 88 | 2.69×10^-3^ |
| Ht | 158.1 | 104 | 5.01×10^-4^ |  | 183.7 | 105 | 3.24×10^-6^ |
| TBil | 18.6 | 20 | 0.548 |  | 19.2 | 19 | 0.444 |
| AST | 80.3 | 69 | 0.165 |  | 115.8 | 68 | 2.69×10^-4^ |
| ALT | 37.3 | 21 | 0.015 |  | 28.1 | 21 | 0.136 |
| ALP | 104.2 | 80 | 0.036 |  | 120.9 | 82 | 3.37×10^-3^ |
| GGT | 129.0 | 91 | 5.44×10^-3^ |  | 128.3 | 93 | 8.93×10^-3^ |
| TP | 129.1 | 83 | 9.00×10^-4^ |  | 114.6 | 85 | 0.018 |
| Alb | 64.7 | 38 | 4.46×10^-3^ |  | 58.1 | 39 | 0.025 |
| sCr | 182.6 | 140 | 9.02×10^-3^ |  | 181.5 | 140 | 0.011 |
| BUN | 78.3 | 52 | 0.011 |  | 77.7 | 51 | 9.31×10^-3^ |
| UA | 69.6 | 56 | 0.104 |  | 71.8 | 55 | 0.064 |
| Ca | 61.3 | 43 | 0.034 |  | 50.6 | 45 | 0.262 |
| P | 27.6 | 27 | 0.430 |  | 50.3 | 27 | 4.15×10^-3^ |

^*^ *df*: degree of freedom (= Number of IVs - 1).

**Table S11.** **Potential bias in UVMR estimates due to sample overlap between GWAS of clinical factors and CAD based on BBJ.**

| **Clinical factor** | **Maximum sample overlap** | **Maximum bias (Proportion)** |
| --- | --- | --- |
| Height | 74.9% | -1.09×10^-2^ (7.2%) |
| BMI | 74.5% | 4.84×10^-2^ (9.4%) |
| FG | 43.8% | 7.57×10^-3^ (2.2%) |
| HbA1c | 20.2% | 4.71×10^-3^ (1.5%) |
| T2D | 90.3% | 1.37×10^-3^ (1.1%) |
| HDL-C | 33.3% | -2.29×10^-3^ (1.9%) |
| LDL-C | 34.3% | 8.03×10^-3^ (1.4%) |
| TG | 49.7% | 2.25×10^-2^ (6.2%) |
| TC | 60.4% | 1.64×10^-2^ (3.2%) |
| SBP | 64.3% | 2.82×10^-2^ (5.4%) |
| DBP | 64.3% | 2.46×10^-2^ (4.2%) |
| CRP | 35.5% | 1.05×10^-2^ (6.9%) |
| WBC | 50.8% | -3.74×10^-3^ (5.6%) |
| Lym | 29.2% | -1.61×10^-2^ (9.3%) |
| Mono | 29.2% | 2.08×10^-2^ (7.8%) |
| Neutro | 29.2% | -6.18×10^-4^ (2.4%) |
| Eosino | 29.2% | -4.83×10^-4^ (1.2%) |
| Baso | 29.2% | 9.72×10^-4^ (1.8%) |
| Plt | 50.9% | -1.46×10^-3^ (2.7%) |
| RBC | 51.2% | 4.45×10^-3^ (2.4%) |
| MCV | 51.0% | -3.63×10^-4^ (1.6%) |
| MCH | 50.9% | -4.17×10^-4^ (1.7%) |
| MCHC | 51.2% | -1.96×10^-4^ (1.8%) |
| Hb | 51.2% | 7.57×10^-3^ (3.1%) |
| Ht | 51.2% | 8.21×10^-3^ (3.1%) |
| TBil | 51.9% | 3.67×10^-5^ (0.1%) |
| AST | 63.1% | -9.59×10^-4^ (10.1%) |
| ALT | 63.2% | -2.74×10^-2^ (7.5%) |
| ALP | 49.4% | -2.54×10^-4^ (2.5%) |
| GGT | 55.7% | 8.55×10^-3^ (6.6%) |
| TP | 53.4% | 1.12×10^-3^ (1.6%) |
| Alb | 48.1% | 3.04×10^-3^ (1.7%) |
| sCr | 66.9% | 8.37×10^-3^ (4.4%) |
| BUN | 65.8% | 2.22×10^-3^ (1.8%) |
| UA | 51.3% | 4.06×10^-3^ (1.7%) |
| Ca | 33.7% | 2.30×10^-4^ (1.7%) |
| P | 20.1% | -1.22×10^-3^ (1.3%) |

The bias was calculated as $\beta r/F$, where $\beta$ was plugged-in with the MR-Corr estimate and $r$ was the sample overlap rate between GWAS of the exposure (study 1) and the outcome (study 1) and $F$ was the mean *F* statistic averaged across IVs. The maximum sample overlap rate was calculated as ${min(n_{1},n_{2})}/{max(n_{1},n_{2})}$, where $n_{1}$ and $n_{2}$ were the sample sizes of study 1 and 2, respectively.

**Table S12. MVMR causal effect estimates of 10 significant clinical factors on CAD after adjusting for 6 cardiometabolic factors.**

| **Clinical factor** | **Population** | **Conditional *F*** | ***OR* (95% CI)** | ***P*** |
| --- | --- | --- | --- | --- |
| Height | East Asian | 14.86 | 0.87 (0.81, 0.92) | 9.75×10^-6^ |
|  | European | 20.82 | 0.90 (0.86, 0.94) | 4.61×10^-7^ |
|  | Meta-analysis | - | 0.89 (0.86, 0.92) | 3.14×10^-11^ |
| BMI | East Asian | 4.21 | 1.23 (1.11, 1.37) | 8.46×10^-5^ |
|  | European | 7.12 | 1.20 (1.13, 1.27) | 2.09×10^-9^ |
|  | Meta-analysis | - | 1.21 (1.15, 1.27) | 8.35×10^-13^ |
| HbA1c | East Asian | 2.19 | 1.20 (1.13, 1.28) | 2.08×10^-8^ |
|  | European | 8.69 | 1.17 (1.10, 1.24) | 1.90×10^-7^ |
|  | Meta-analysis | - | 1.18 (1.13, 1.23) | 2.60×10^-14^ |
| LDL-C | East Asian | 1.46 | 1.23 (1.13, 1.34) | 3.77×10^-6^ |
|  | European | 4.15 | 1.11 (0.95, 1.29) | 0.179 |
|  | Meta-analysis | - | 1.20 (1.11, 1.29) | 3.03×10^-6^ |
| TG | East Asian | 1.63 | 1.30 (1.11, 1.51) | 1.16×10^-3^ |
|  | European | 2.88 | 1.17 (1.04, 1.32) | 8.97×10^-3^ |
|  | Meta-analysis | - | 1.22 (1.11, 1.34) | 5.23×10^-5^ |
| SBP | East Asian | 1.99 | 1.34 (1.20, 1.51) | 3.95×10^-7^ |
|  | European | 4.53 | 1.55 (1.42, 1.70) | 4.28×10^-21^ |
|  | Meta-analysis | - | 1.47 (1.37, 1.58) | 6.51×10^-26^ |
| RBC | East Asian | 2.76 | 1.09 (1.02, 1.16) | 0.013 |
|  | European | 8.98 | 1.06 (0.98, 1.14) | 0.122 |
|  | Meta-analysis | - | 1.07 (1.02, 1.13) | 4.09×10^-3^ |
| Hb | East Asian | 1.94 | 1.04 (0.97, 1.13) | 0.274 |
|  | European | 5.48 | 1.19 (1.08, 1.31) | 3.52×10^-4^ |
|  | Meta-analysis | - | 1.10 (1.03, 1.16) | 2.01×10^-3^ |
| Ht | East Asian | 2.05 | 1.07 (0.99, 1.15) | 0.080 |
|  | European | 5.97 | 1.16 (1.05, 1.28) | 2.64×10^-3^ |
|  | Meta-analysis | - | 1.10 (1.04, 1.17) | 1.24×10^-3^ |
| UA | East Asian | 4.79 | 1.12 (1.06, 1.19) | 3.26×10^-5^ |
|  | European | 11.48 | 1.00 (0.95, 1.06) | 0.953 |
|  | Meta-analysis | - | 1.06 (1.02, 1.11) | 2.25×10^-3^ |

**
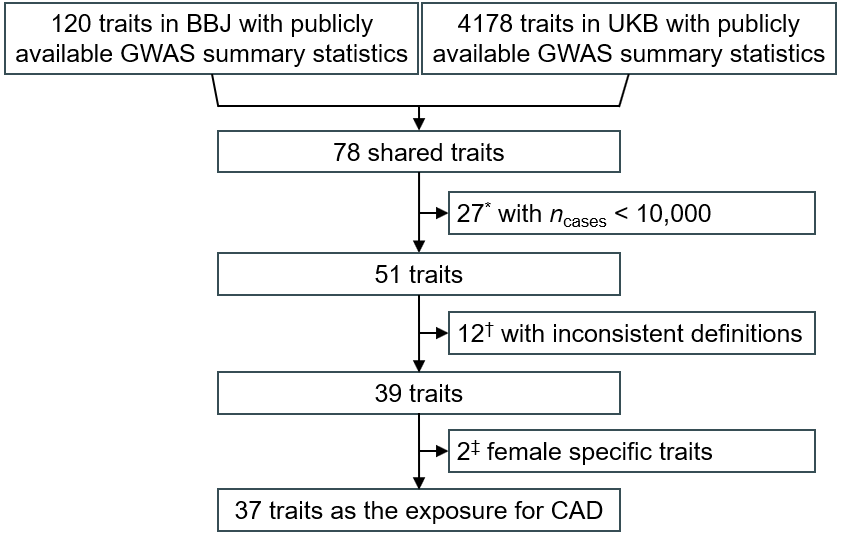
**

**Fig S1. Selection procedure of 37 clinical factors for MR analyses.**

^*^ 27 diseases with case sample size < 10,000: arrhythmia, asthma, atrial fibrillation, breast cancer, cerebral aneurysm, cervical cancer, chronic obstructive pulmonary disease, cirrhosis, colorectal cancer, endometrial cancer, endometriosis, epilepsy, esophageal cancer, glaucoma, ischemic stroke, lung cancer, interstitial lung disease, periodontal disease, primary open-angle glaucoma, peripheral artery disease, prostate cancer, pulmonary tuberculosis, rheumatoid arthritis, osteoporosis, ovarian cancer, urolithiasis, uterine fibroids.

^†^ 12 traits with inconsistent definition between BBJ and UKB: cataract, smoking behavior, drinking behavior, 7 dietary habits (coffee, cheese, fish, meat, milk, vegetables, yogurt), 2 electrolyte biomarkers (potassium and sodium).

^‡^ 2 female specific traits: age at menarche, age at natural menopause.

**
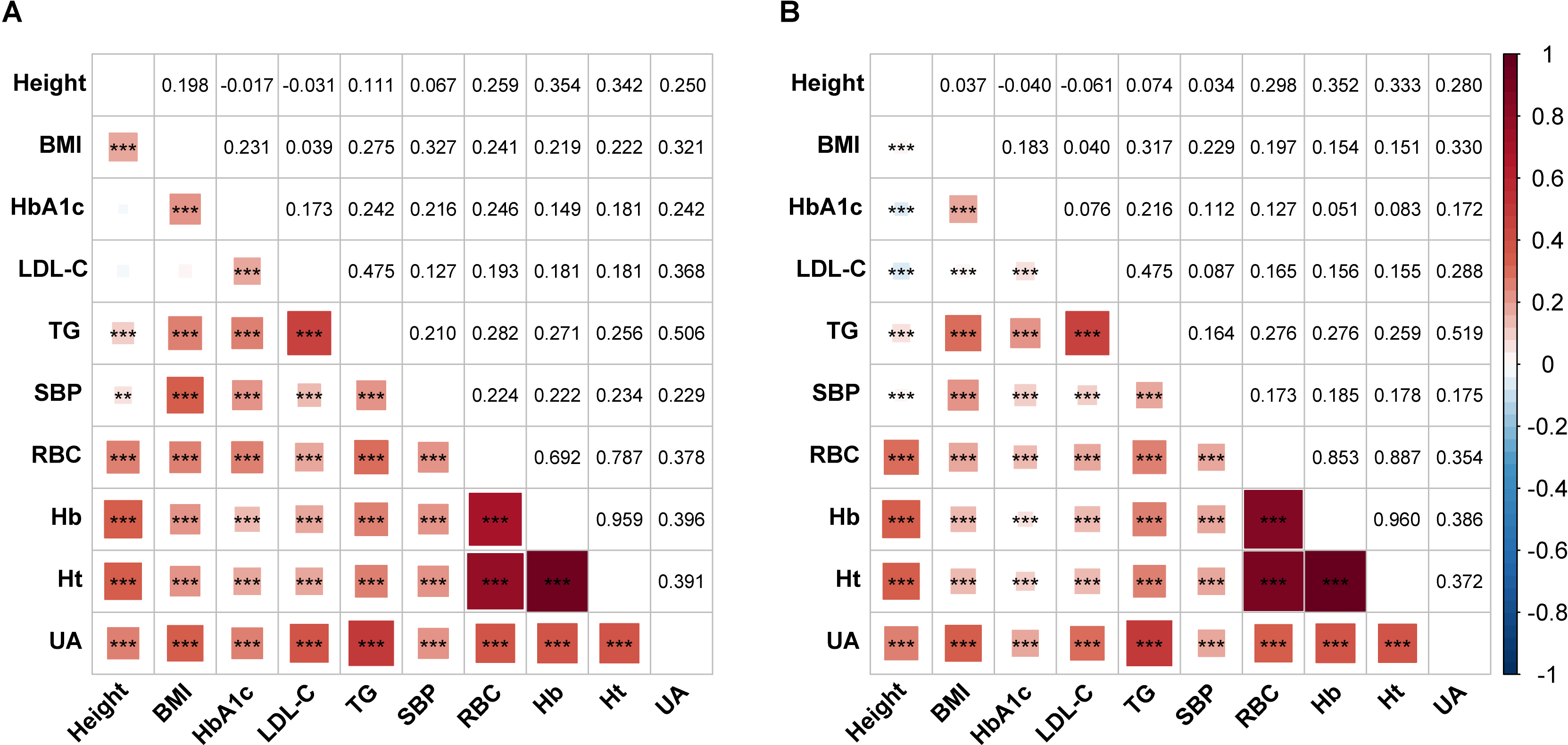
**

**Fig S2. Phenotypic correlation between 10 significant clinical factors in the MVMR analyses. (A)** Correlation in East Asians based on 1,575 Chinese in UKB. **(B)** Correlation in Europeans based on 472,671 white-British in UKB. We calculated the Pearson correlation between traits after inverse-rank normalization of the individual data. Numerical values are shown in the upper triangle, and the statistical significance is indicated in the lower triangle: *, *P* < 0.05; **, *P* < 0.01; ***, *P* < 0.001.


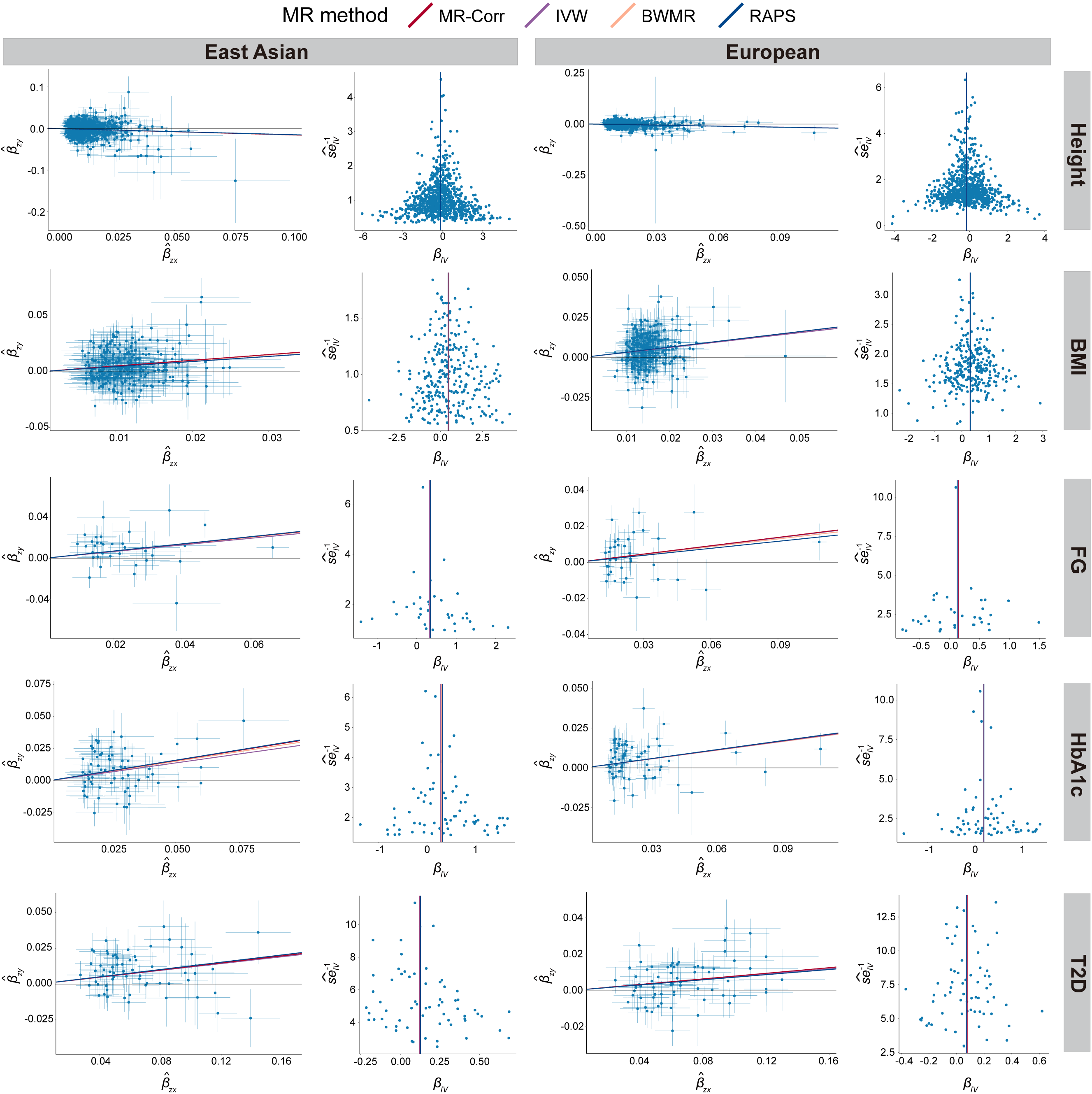


**Fig S3. Scatter plot and funnel plot for each exposure (Height, BMI, FG, HbA1c, T2D) in the MR analyses in East Asians and Europeans.** In each scatter plot (left), each dot represents an IV, with the x and y axes being the genetic associations with the exposure ($\hat{\beta}_{zx}$) and the outcome ($\hat{\beta}_{zy}$), respectively, and the bars represent one standard error. The funnel plot (right) displays the estimated causal effect based on each IV ($\hat{\beta}_{IV}$) and the reciprocal of its standard error ($\hat{se}_{IV}^{-1}$). Colored solid lines illustrate the estimated causal effects of the exposure on CAD, combining across all IVs.

**
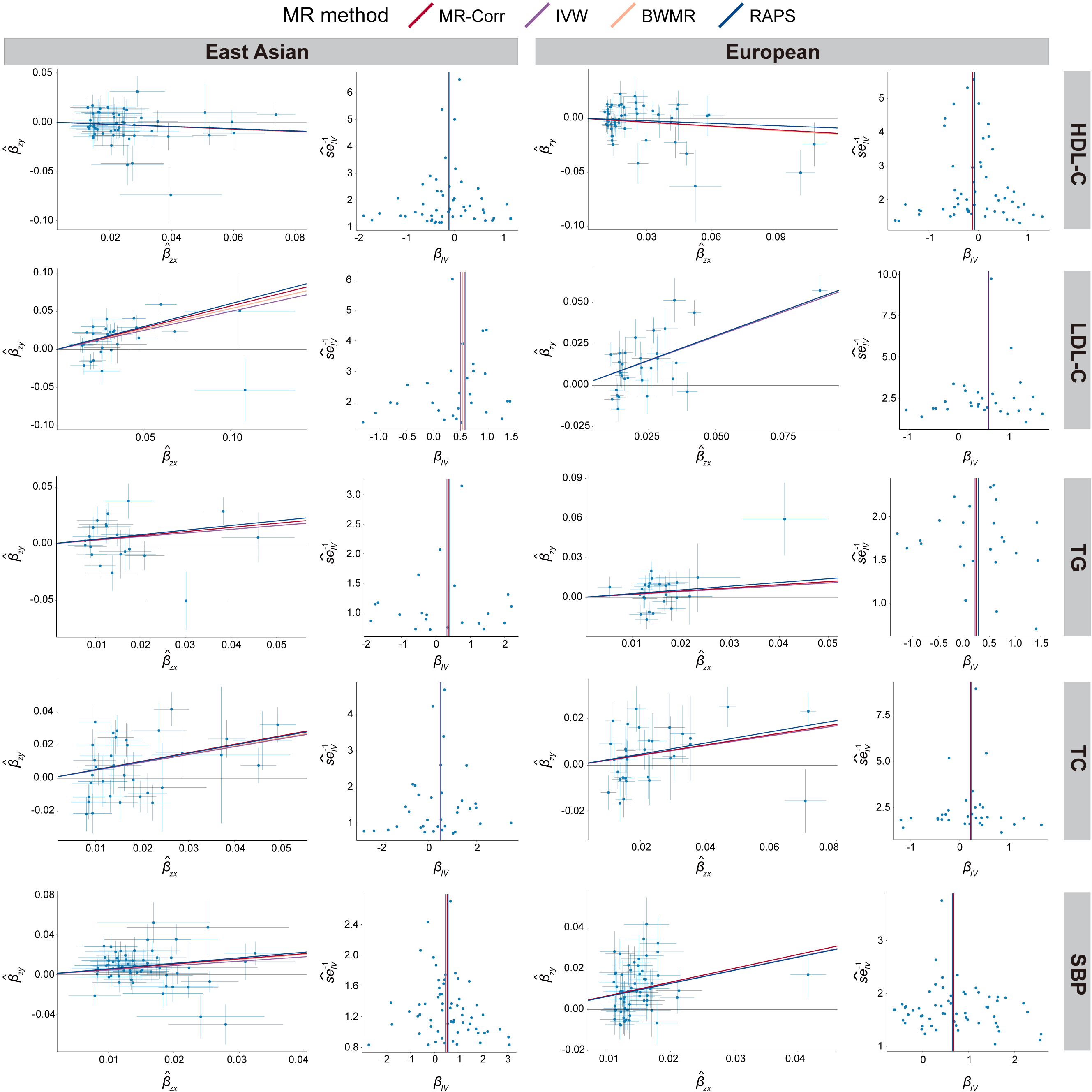
**

**Fig S4. Scatter plot and funnel plot for each exposure (HDL-C, LDL-C, TG, TC, SBP) in the MR analyses in East Asians and Europeans.** In each scatter plot (left), each dot represents an IV, with the x and y axes being the genetic associations with the exposure ($\hat{\beta}_{zx}$) and the outcome ($\hat{\beta}_{zy}$), respectively, and the bars represent one standard error. The funnel plot (right) displays the estimated causal effect based on each IV ($\hat{\beta}_{IV}$) and the reciprocal of its standard error ($\hat{se}_{IV}^{-1}$). Colored solid lines illustrate the estimated causal effects of the exposure on CAD, combining across all IVs.

**
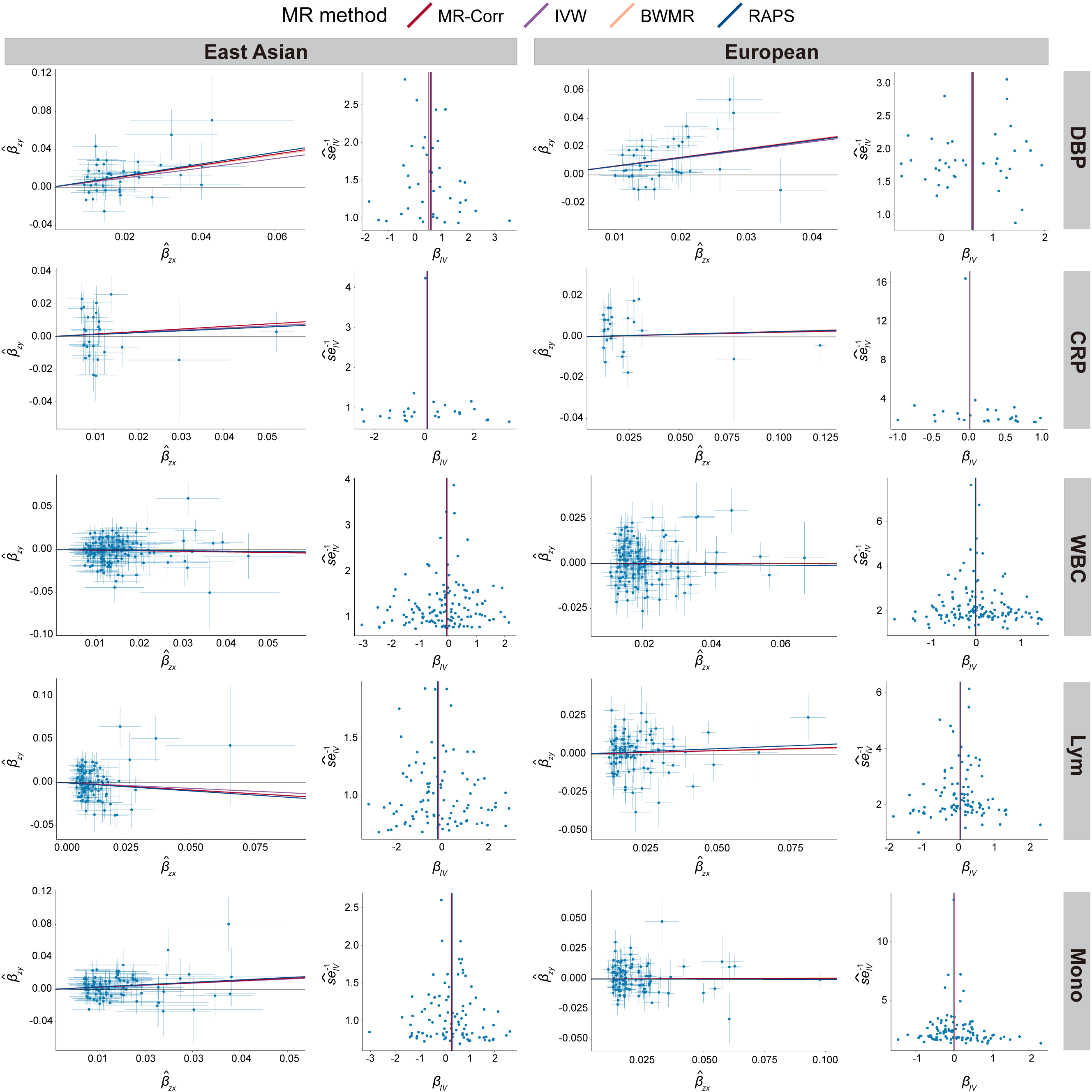
**

**Fig S5. Scatter plot and funnel plot for each exposure (DBP, CRP, WBC, Lym, Mono) in the MR analyses in East Asians and Europeans.** In each scatter plot (left), each dot represents an IV, with the x and y axes being the genetic associations with the exposure ($\hat{\beta}_{zx}$) and the outcome ($\hat{\beta}_{zy}$), respectively, and the bars represent one standard error. The funnel plot (right) displays the estimated causal effect based on each IV ($\hat{\beta}_{IV}$) and the reciprocal of its standard error ($\hat{se}_{IV}^{-1}$). Colored solid lines illustrate the estimated causal effects of the exposure on CAD, combining across all IVs.

**
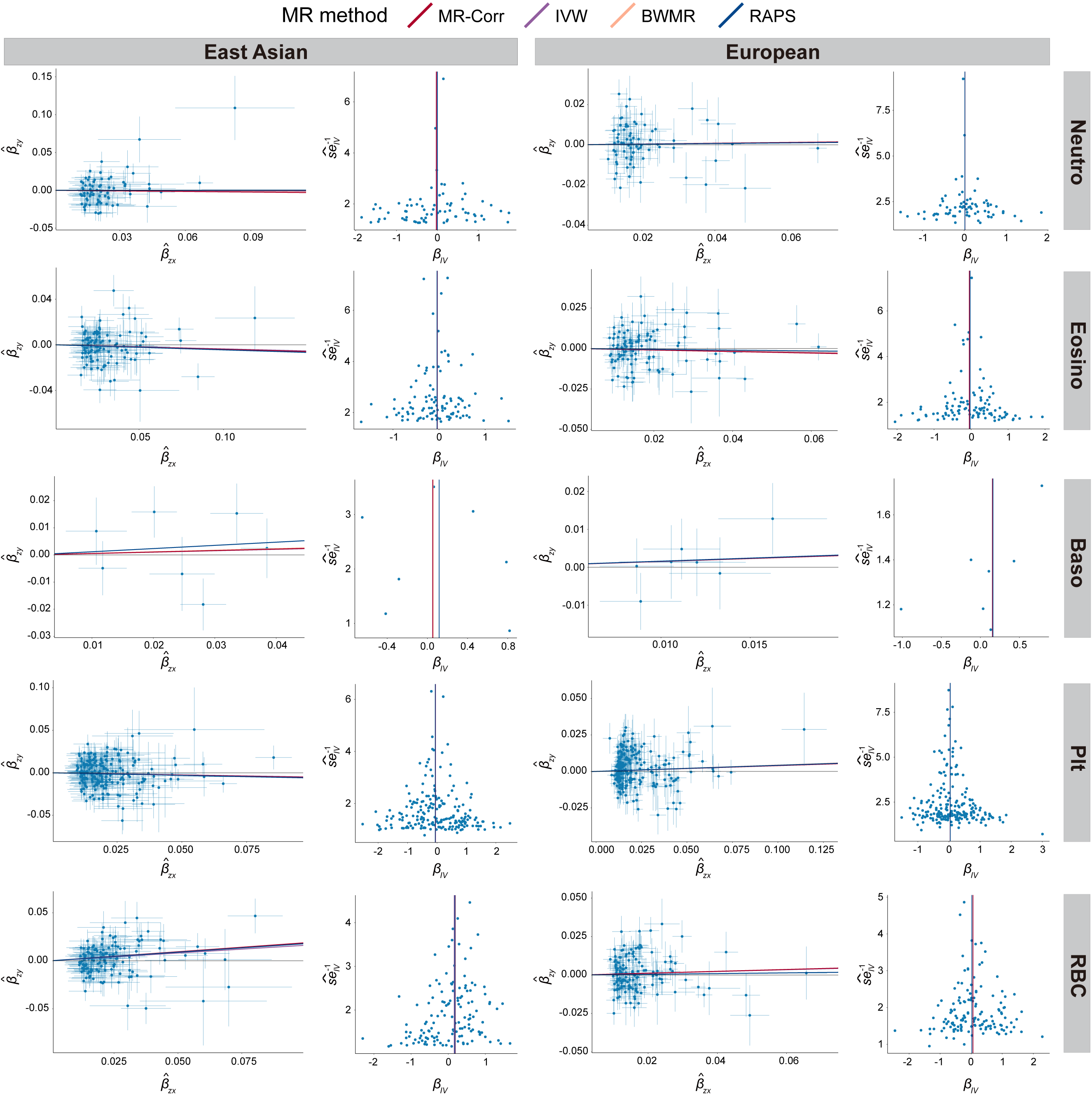
**

**Fig S6. Scatter plot and funnel plot for each exposure (Neutro, Eosino, Baso, Plt, RBC) in the MR analyses in East Asians and Europeans.** In each scatter plot (left), each dot represents an IV, with the x and y axes being the genetic associations with the exposure ($\hat{\beta}_{zx}$) and the outcome ($\hat{\beta}_{zy}$), respectively, and the bars represent one standard error. The funnel plot (right) displays the estimated causal effect based on each IV ($\hat{\beta}_{IV}$) and the reciprocal of its standard error ($\hat{se}_{IV}^{-1}$). Colored solid lines illustrate the estimated causal effects of the exposure on CAD, combining across all IVs.

**
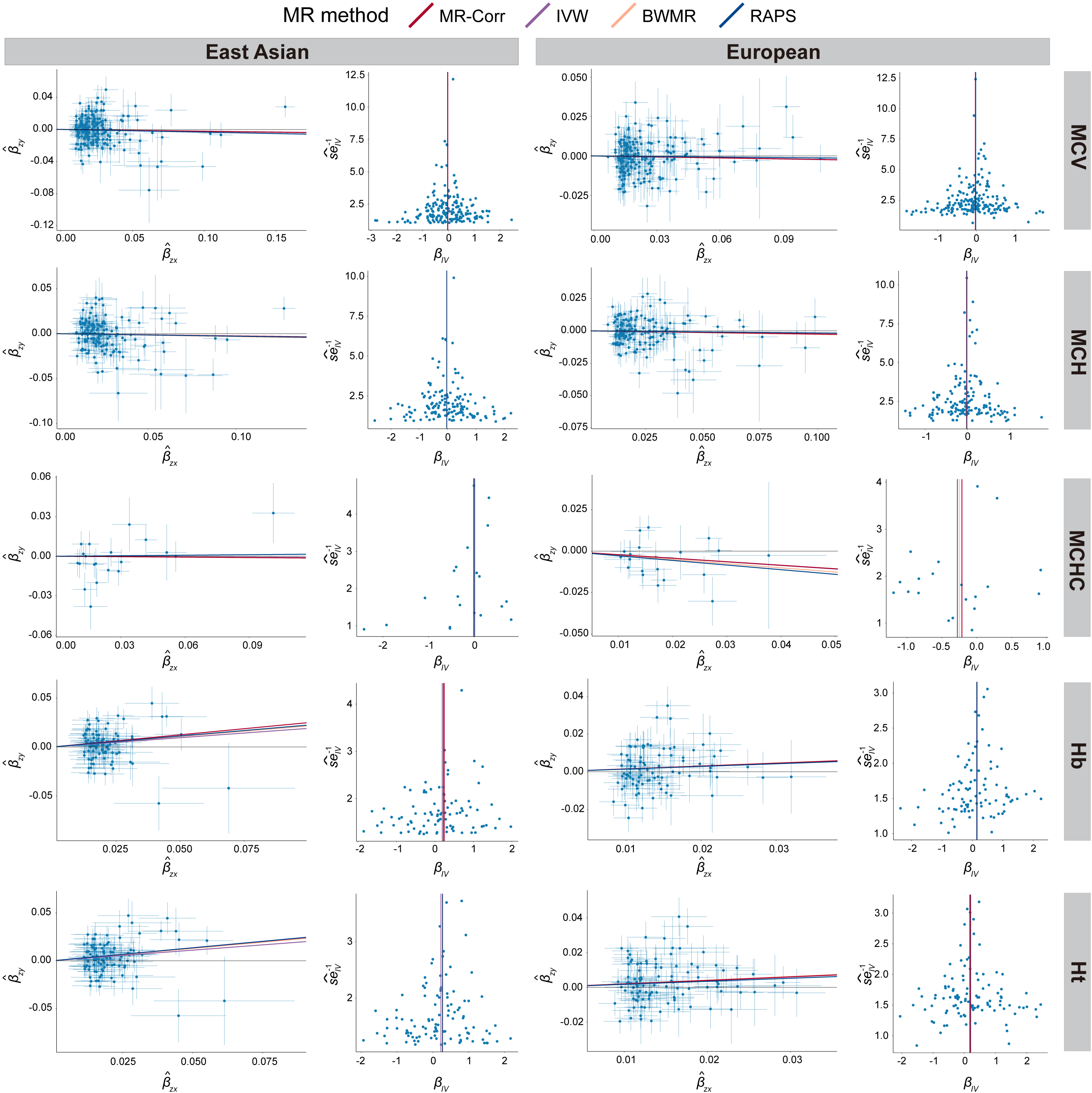
**

**Fig S7. Scatter plot and funnel plot for each exposure (MCV, MCH, MCHC, Hb, Ht) in the MR analyses in East Asians and Europeans.** In each scatter plot (left), each dot represents an IV, with the x and y axes being the genetic associations with the exposure ($\hat{\beta}_{zx}$) and the outcome ($\hat{\beta}_{zy}$), respectively, and the bars represent one standard error. The funnel plot (right) displays the estimated causal effect based on each IV ($\hat{\beta}_{IV}$) and the reciprocal of its standard error ($\hat{se}_{IV}^{-1}$). Colored solid lines illustrate the estimated causal effects of the exposure on CAD, combining across all IVs.

_
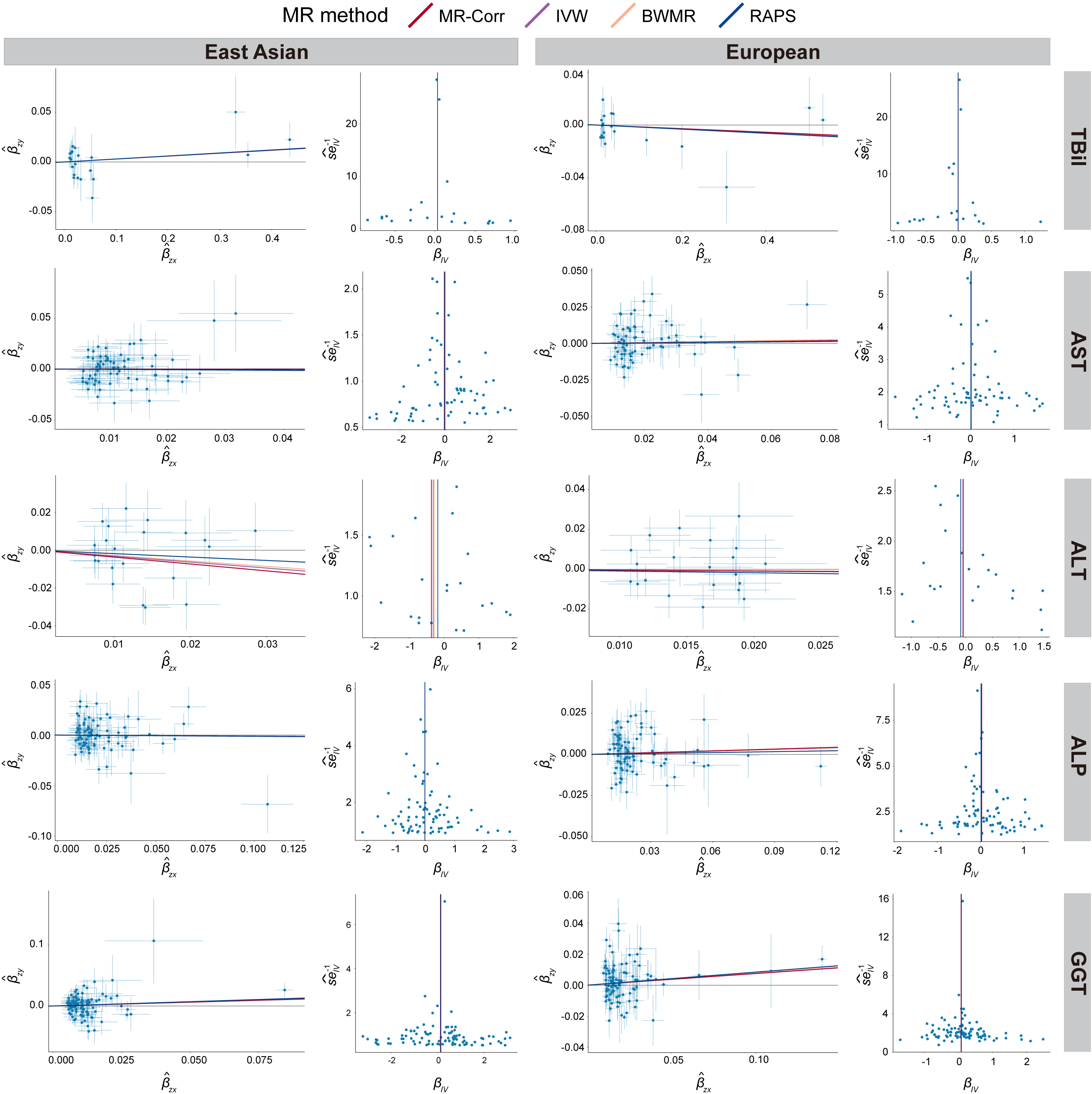
_

**Fig S8. Scatter plot and funnel plot for each exposure (TBil, AST, ALT, ALP, GGT) in the MR analyses in East Asians and Europeans.** In each scatter plot (left), each dot represents an IV, with the x and y axes being the genetic associations with the exposure ($\hat{\beta}_{zx}$) and the outcome ($\hat{\beta}_{zy}$), respectively, and the bars represent one standard error. The funnel plot (right) displays the estimated causal effect based on each IV ($\hat{\beta}_{IV}$) and the reciprocal of its standard error ($\hat{se}_{IV}^{-1}$). Colored solid lines illustrate the estimated causal effects of the exposure on CAD, combining across all IVs.

**
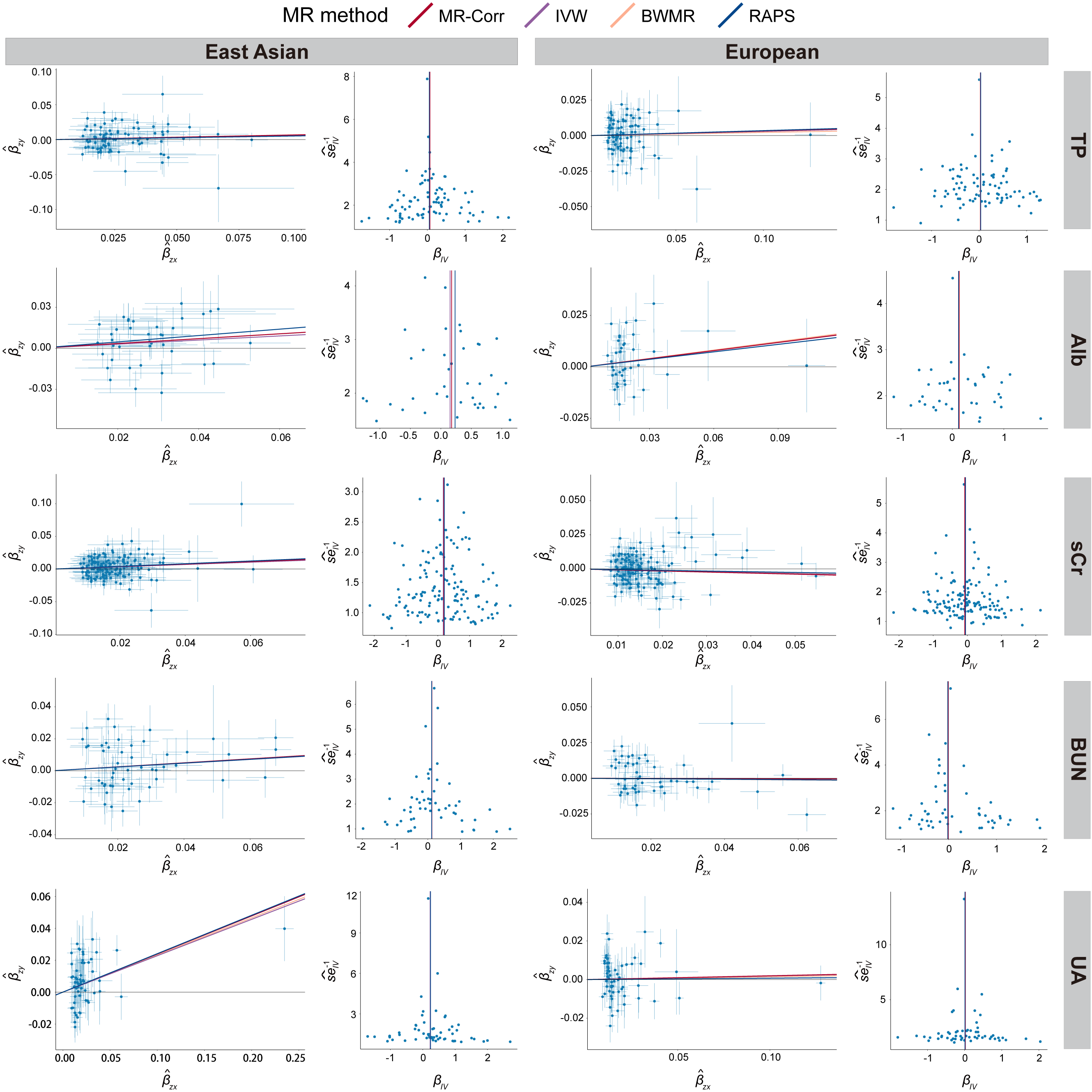
**

**Fig S9. Scatter plot and funnel plot for each exposure (TP, Alb, sCr, BUN, UA) in the MR analyses in East Asians and Europeans.** In each scatter plot (left), each dot represents an IV, with the x and y axes being the genetic associations with the exposure ($\hat{\beta}_{zx}$) and the outcome ($\hat{\beta}_{zy}$), respectively, and the bars represent one standard error. The funnel plot (right) displays the estimated causal effect based on each IV ($\hat{\beta}_{IV}$) and the reciprocal of its standard error ($\hat{se}_{IV}^{-1}$). Colored solid lines illustrate the estimated causal effects of the exposure on CAD, combining across all IVs.

**
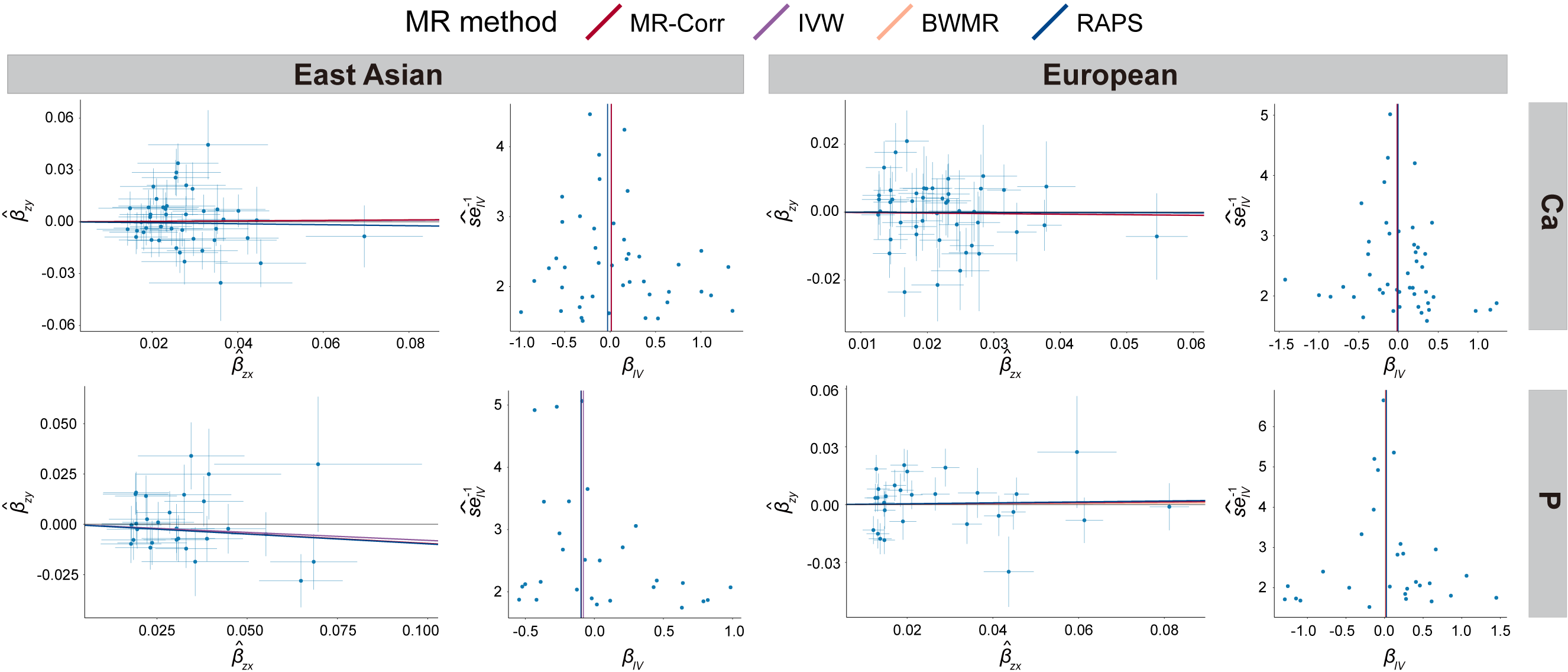
**

**Fig S10. Scatter plot and funnel plot for each exposure (Ca, P) in the MR analyses in East Asians and Europeans.** In each scatter plot (left), each dot represents an IV, with the x and y axes being the genetic associations with the exposure ($\hat{\beta}_{zx}$) and the outcome ($\hat{\beta}_{zy}$), respectively, and the bars represent one standard error. The funnel plot (right) displays the estimated causal effect based on each IV ($\hat{\beta}_{IV}$) and the reciprocal of its standard error ($\hat{se}_{IV}^{-1}$). Colored solid lines illustrate the estimated causal effects of the exposure on CAD, combining across all IVs.
