# Supplementary material for: Mendelian randomization analysis of 37 clinical factors and coronary artery disease in East Asian and European populations
Source: Genome Med. 2022 Jun 14;14:63. doi: 10.1186/s13073-022-01067-1 (PMC9195360; doi:10.1186/s13073-022-01067-1)
Supplement: Supplementary file 3 — Additional file 3. STROBE-MR-checklist. [file 13073_2022_1067_MOESM3_ESM.docx]

**STROBE-MR checklist of recommended items to address in reports of Mendelian randomization studies**1 2

| **Item No.** | **Section** | **Checklist item** | **Page No.** | **Relevant text from manuscript or supplements** |
| --- | --- | --- | --- | --- |
| 1 | **TITLE and ABSTRACT** | Indicate Mendelian randomization (MR) as the study’s design in the title and/or the abstract if that is a main purpose of the study | 1-3 | Title: “Mendelian randomization analysis of 37 clinical factors and coronary artery disease in East Asian and European populations”  Abstract: “We performed univariable and multivariable Mendelian randomization (MR) analyses in East Asians and Europeans separately, followed by meta-analysis” |
|  | **INTRODUCTION** |  |  |  |
| 2 | **Background** | Explain the scientific background and rationale for the reported study. What is the exposure? Is a potential causal relationship between exposure and outcome plausible? Justify why MR is a helpful method to address the study question | 4 | “Improved understanding of the causality and effect sizes of different clinical factors can …… for CAD.”  “To date, although hundreds of risk factors being reported to associate with CAD by epidemiological studies, causal inference of these associations was hindered …...”  “The recent advances of large-scale genome-wide association studies (GWAS) and Mendelian randomization (MR) methods have enabled evaluation of the causality between risk factors and disease outcomes” |
| 3 | **Objectives** | State specific objectives clearly, including pre-specified causal hypotheses (if any). State that MR is a method that, under specific assumptions, intends to estimate causal effects | 5 | “……we investigated and compared the causal effects of 37 clinical factors on CAD in East Asian and European populations.” |
|  | **METHODS** |  |  |  |
| 4 | **Study design and data sources** | Present key elements of the study design early in the article. Consider including a table listing sources of data for all phases of the study. For each data source contributing to the analysis, describe the following: |  |  |
|  | a) | Setting: Describe the study design and the underlying population, if possible. Describe the setting, locations, and relevant dates, including periods of recruitment, exposure, follow-up, and data collection, when available. | 5-8 | “For East Asians, we collected summary statistics of clinical factors from BBJ, a patient-based biobank with ~200,000 participants recruited from 12 medical institutions across Japan in 2003-2008”  “The European GWAS of clinical factors were based UKB, a population cohort with over 500,000 participants recruited from 22 assessment centers throughout the UK in 2006-2010”  “Figure 1 illustrates the overall study design……”  “We first identified 78 traits on common across the BBJ and UKB GWAS databases……” |
|  | b) | Participants: Give the eligibility criteria, and the sources and methods of selection of participants. Report the sample size, and whether any power or sample size calculations were carried out prior to the main analysis | 5-6 |  |
|  | c) | Describe measurement, quality control and selection of genetic variants | - |  |
|  | d) | For each exposure, outcome, and other relevant variables, describe methods of assessment and diagnostic criteria for diseases | 6 | “Summary statistics of CAD in East Asians ……, consisting of 29,319 cases and 183,134 controls primarily from BBJ. CAD in Ishigaki et al. 17 included physician-diagnosed stable angina, unstable angina, and myocardial infarction (MI).”  “For Europeans, we meta-analyzed CAD GWAS summary statistics from the CARDIoGRAMplusC4D consortium and the FinnGen study….…with CAD determined by the International Classification of Diseases version 10 (ICD-10), including angina (I20), MI (I21, I22), complications following MI (I23), status post-acute MI (I253), coronary atherosclerosis (I24, I25, Z951, T822), and coronary revascularization……in which CAD included chronic stable angina, MI, acute coronary syndrome, and coronary stenosis >50%.” |
|  | e) | Provide details of ethics committee approval and participant informed consent, if relevant | 23 | “Our analyses were based on publicly available data that have been approved by relevant review boards. No additional ethical review or informed consent was sought.” |
| 5 | **Assumptions** | Explicitly state the three core IV assumptions for the main analysis (relevance, independence and exclusion restriction) as well assumptions for any additional or sensitivity analysis | 8 | “Valid IVs need to be associated with the exposure (the relevance assumption), have no association with any confounders (the independence assumption), and have no association with the outcome conditional on the exposure (the exclusion restriction assumption)” |
| 6 | **Statistical methods: main analysis** | Describe statistical methods and statistics used |  |  |
|  | a) | Describe how quantitative variables were handled in the analyses (i.e., scale, units, model) | 10 | “We calculated the odds ratio (OR) and the corresponding 95% confidence interval (CI) of CAD per SD increment of a quantitative exposure……. SDs for quantitative traits were presented in Supplementary Tables 2-3, in which the values for UKB (SD_UKB_) were calculated using individual phenotype data of 472,671 white-British participants and the values for BBJ (SD_BBJ_) were obtained from reference 16. ……wrescaled the SNP effect sizes (and standard errors) of IVs from BBJ by SD_UKB_/SD_BBJ_, such that the MR causal effect estimates from two populations are in the same unit of SD_UKB_.” |
|  | b) | Describe how genetic variants were handled in the analyses and, if applicable, how their weights were selected | 8-9 | “Assuming causal variants were largely shared between populations 26, we selected one set of IVs for both populations while using population-specific SNP effect sizes for the MR analyses in either population.” |
|  | c) | Describe the MR estimator (e.g. two-stage least squares, Wald ratio) and related statistics. Detail the included covariates and, in case of two-sample MR, whether the same covariate set was used for adjustment in the two samples | 9-10 | “Four UVMR methods were utilized: the MR-Corr method, the IVW method, the Bayesian weighted Mendelian randomization (BWMR) method, and the robust adjusted profile score (RAPS) method.” |
|  | d) | Explain how missing data were addressed | - |  |
|  | e) | If applicable, indicate how multiple testing was addressed | 10 | “Bonferroni-corrected thresholds (0.05/37 = 0.00135 in the forward MR and 0.05/4 = 0.0125 in the reverse MR) were adopted to account for multiple testing.” |
| 7 | **Assessment of assumptions** | Describe any methods or prior knowledge used to assess the assumptions or justify their validity | 11 | “To evaluate the validity of UVMR analyses, we computed the proportion of variance explained by each IV (*PVE*) and the corresponding F statistic……” |
| 8 | **Sensitivity analyses and additional analyses** | Describe any sensitivity analyses or additional analyses performed (e.g. comparison of effect estimates from different approaches, independent replication, bias analytic techniques, validation of instruments, simulations) | 9-12 | “Four UVMR methods were utilized: the MR-Corr method, the IVW method, the Bayesian weighted Mendelian randomization (BWMR) method, and the robust adjusted profile score (RAPS) method.”  “Besides, we estimated the potential bias introduced by sample overlap……”  “We then performed MVMR analyses for each of red blood cell count (RBC), hemoglobin (Hb), hematocrit (Ht) and uric acid (UA), adjusting for the aforementioned 6 cardiometabolic factors.” |
| 9 | **Software and pre- registration** |  |  |  |
|  | a) | Name statistical software and package(s), including version and settings used | 10 | “MR analyses were conducted using the MR.Corr2, TwoSampleMR, BWMR, and mr.raps R packages.” |
|  | b) | State whether the study protocol and details were pre-registered (as well as when and where) | - |  |
|  | **RESULTS** |  |  |  |
| 10 | **Descriptive data** |  |  |  |
|  | a) | Report the numbers of individuals at each stage of included studies and reasons for exclusion. Consider use of a flow diagram | - |  |
|  | b) | Report summary statistics for phenotypic exposure(s), outcome(s), and other relevant variables (e.g. means, SDs, proportions) |  | Supplementary Table 2 & 3 |
|  | c) | If the data sources include meta-analyses of previous studies, provide the assessments of heterogeneity across these studies | - |  |
|  | d) | For two-sample MR:  i. Provide justification of the similarity of the genetic variant-exposure associations between the exposure and outcome samples  ii. Provide information on the number of individuals who overlap between the exposure and outcome studies |  | Supplementary Table 11 |
| 11 | **Main results** |  |  |  |
|  | a) | Report the associations between genetic variant and exposure, and between genetic variant and outcome, preferably on an interpretable scale | - |  |
|  | b) | Report MR estimates of the relationship between exposure and outcome, and the measures of uncertainty from the MR analysis, on an interpretable scale, such as odds ratio or relative risk per SD difference | 13-16 | “By UVMR analyses, we identified 14 significant risk factors: four in East Asians only, one in meta-analysis only, and nine in both populations……”  “For 13 hematological indices, we found evidence of causal effects on CAD for RBC (*OR* = 1.21 per 40.94×10^4^/μL increment, 1.10-1.33, *P* = 9.46×10^-5^), Hb (1.28 per 1.23 g/dL increment, 1.11-1.47, *P* = 4.87×10^-4^), and Ht (1.31 per 3.53% increment, 1.16-1.48, *P* = 1.18×10^-5^) in East Asians, with similar results yielded by different MR methods……” |
|  | c) | If relevant, consider translating estimates of relative risk into absolute risk for a meaningful time period | - |  |
|  | d) | Consider plots to visualize results (e.g. forest plot, scatterplot of associations between genetic variants and outcome versus between genetic variants and exposure) |  | Figure 3-5, Supplementary Figures 3-10 |
| 12 | **Assessment of assumptions** |  |  |  |
|  | a) | Report the assessment of the validity of the assumptions | 14-15 | “We confirmed the validity of our UVMR analyses from three aspects. First, the mean F statistics for the valid IVs were all above 10……” |
|  | b) | Report any additional statistics (e.g., assessments of heterogeneity across genetic variants, such as I2, Q statistic or E-value) | 15 | “Second, although Cochran’s Q tests suggested heterogeneity in the causal estimates for some clinical factors (*P*_het_ < 0.01 in Supplementary Table 10), we observed no obvious directional horizontal pleiotropy in the funnel plots (Supplementary Figures 3-10)” |
| 13 | **Sensitivity analyses and additional analyses** |  |  |  |
|  | a) | Report any sensitivity analyses to assess the robustness of the main results to violations of the assumptions | 15 | “Second, although Cochran’s Q tests suggested heterogeneity in the causal estimates for some clinical factors (*P*_het_ < 0.01 in Supplementary Table 10), we observed no obvious directional horizontal pleiotropy in the funnel plots (Supplementary Figures 3-10)” |
|  | b) | Report results from other sensitivity analyses or additional analyses | 15-16 | “In the MVMR analysis including six cardiometabolic factors (Figure 5A, Supplementary Table 12), all factors showed consistent and independent causal effects on CAD in East Asians and Europeans……” |
|  | c) | Report any assessment of direction of causal relationship (e.g., bidirectional MR) | 13-14 | “Reversely, we found no evidence of causal effect of CAD on RBC (*P*_meta_ = 0.051) or Ht (*P*_meta_ = 0.448, Table 2). In contrast, CAD had significant causal effect on Hb (*P*_meta_ = 2.46×10^-3^), indicating bidirectional causal relationships, which were further supported by Steiger’s directionality test (Supplementary Table 9).” |
|  | d) | When relevant, report and compare with estimates from non-MR analyses | - |  |
|  | e) | Consider additional plots to visualize results (e.g., leave-one-out analyses) | - |  |
|  | **DISCUSSION** |  |  |  |
| 14 | **Key results** | Summarize key results with reference to study objectives | 16 | “By analyzing large GWAS datasets under a unified MR framework, we identified 1 protective and 13 risk factors, the majority of which showed consistent effects between populations. These findings might inform prevention strategies and suggest potential therapeutic targets of CAD.” |
| 15 | **Limitations** | Discuss limitations of the study, taking into account the validity of the IV assumptions,  other sources of potential bias, and imprecision. Discuss both direction and magnitude of any potential bias and any efforts to address them | 20 | “Nonetheless, there are several limitations of our study……” |
| 16 | **Interpretation** |  |  |  |
|  | a) | Meaning: Give a cautious overall interpretation of results in the context of their limitations and in comparison with other studies | 18-19 | “While 35 of the 37 clinical factors examined in our study have been reported to associate with CAD by epidemiological studies……” |
|  | b) | Mechanism: Discuss underlying biological mechanisms that could drive a potential causal relationship between the investigated exposure and the outcome, and whether the gene-environment equivalence assumption is reasonable. Use causal language carefully, clarifying that IV estimates may provide causal effects only under certain assumptions | 16-17 | “Our results highlight causal effects of red blood cell traits, including RBC, hemoglobin, and hematocrit, independent of traditional cardiometabolic factors……” |
|  | c) | Clinical relevance: Discuss whether the results have clinical or public policy relevance, and to what extent they inform effect sizes of possible interventions | 16-17 | “……we confirmed the causal role of RBC on CAD, elevating the risk by nearly 10% per SD increase in RBC, hemoglobin, or hematocrit, even after adjusting for cardiometabolic factors……” |
| 17 | **Generalizability** | Discuss the generalizability of the study results (a) to other populations, (b) across other exposure periods/timings, and (c) across other levels of exposure | 19-20 | “……our analyses are well powered by leveraging largest publicly available GWAS datasets.……”  “In addition, there are concerns about the representativeness of BBJ and UKB to the general population. Nevertheless, it has been pointed out that sufficiently large sample size with different levels of exposures is essential for the generalizability of associations between exposures and diseases, and that the risk factor association results based on UKB are highly consistent to those from nationally representative cohorts” |
|  | **OTHER INFORMATION** |  |  |  |
| 18 | **Funding** | Describe sources of funding and the role of funders in the present study and, if applicable, sources of funding for the databases and original study or studies on which the present study is based | 23 | “This study was funded by Natural Science Foundation of China (81973148, 82003561, and 82021005).” |
| 19 | **Data and data sharing** | Provide the data used to perform all analyses or report where and how the data can be accessed, and reference these sources in the article. Provide the statistical code needed to reproduce the results in the article, or report whether the code is publicly accessible and if so, where | 23 | The GWAS summary data of 37 clinical factors were downloaded from BBJ (https://humandbs.biosciencedbc.jp/en/hum0014-v24) and …… |
| 20 | **Conflicts of Interest** | All authors should declare all potential conflicts of interest | 23 | The authors declare that they have no competing interests. |

This checklist is copyrighted by the Equator Network under the Creative Commons Attribution 3.0 Unported (CC BY 3.0) license.

1. Skrivankova VW, Richmond RC, Woolf BAR, Yarmolinsky J, Davies NM, Swanson SA, et al. Strengthening the Reporting of Observational Studies in Epidemiology using Mendelian Randomization (STROBE-MR) Statement. JAMA. 2021;under review.
2. Skrivankova VW, Richmond RC, Woolf BAR, Davies NM, Swanson SA, VanderWeele TJ, et al. Strengthening the Reporting of Observational Studies in Epidemiology using Mendelian Randomisation (STROBE-MR): Explanation and Elaboration. BMJ. 2021;375:n2233.
